# MCJ modulates mitochondrial ETC flux to promote lipid metabolism–driven enhancement of cell proliferation and migration

**DOI:** 10.1038/s41419-025-08398-y

**Published:** 2026-01-02

**Authors:** Priyadarshika Pradhan, Tanvi Chaudhary, Shivali Mishra, Peter Konik, Eva Durinova, Roman Tuma, Abhijt De, Devanjan Sinha

**Affiliations:** 1 Department of Zoology, Institute of Science, Banaras Hindu University, Varanasi, India; 2 Advanced Center for Treatment Research and Education in Cancer (ACTREC), Navi Mumbai, India; 3 Faculty of Science, University of South Bohemia, České Budějovice, Czech Republic

**Keywords:** Cancer metabolism, Energy metabolism

## Abstract

Mitochondrial metabolism plays a crucial role in cancer progression and is associated with effective channeling of electrons through Complex I. The ability to adapt this electron flow as per cellular demands is critical for energy homeostasis. Our observations suggest that proliferating cells regulate the electron entry point through alterations in the levels of Methylation-Controlled J-protein (MCJ). Elevated MCJ levels were found to promote aggressive proliferative and migratory phenotypes, leading to increased primary tumor burden. The phenotype was attributed to MCJ-mediated regulation of mitochondrial bioenergetic plasticity, enabling a preferential rerouting of electron flux through succinate dehydrogenase complex (Complex II). Consequently, cells exhibited suppressed glycolysis and a metabolic shift toward lipid-fueled mitochondrial respiration, marked by increased lipid accumulation and its oxidation. Despite Complex I uncoupling, these cells maintained better respiratory output and preserved NADH levels to support an increased redox potential. These findings decouple the reliance on Complex I for effective mitochondrial respiration and underscore the significance of Complex II-driven metabolism in tumor growth, an important consideration for development of future therapeutics, particularly when current strategies predominantly target Complex I-dependent respiration.

## Introduction

Metabolic reprogramming in cancerous cells have been established to be pivotal for sustenance of cellular biogenesis, reproduction and metastasis [1, 2]. The “Warburg effect” first suggested that the upregulation of glycolysis with “irreversible injury to respiration” meet these increased metabolic requisites in cancer cells [3]. However, the modern notion of the “Warburg effect” is confined to upregulation of “aerobic glycolysis” with widely accepted “reverse Warburg effect” indicating that tumour cells mitochondria are fully functional without any compromise in ATP production [4–6]. Although, mutations in mtDNA-encoded catalytic subunits of mitochondrial respiratory complexes (MRCs) and tricarboxylic acid (TCA) cycle enzymes are common in cancer cells, they do not inactivate mitochondrial energy metabolism but rather alter the mitochondrial bioenergetics and biosynthetic state [7, 8]. Today, the concept of metabolic adaptation in tumours often converge on the ability of tumour cells to rewire their metabolism by dynamically restructuring the flux across different pathways like TCA cycle, or fatty-acid metabolism, to harmonize NAD+ recharge with ATP generation [2, 9–12]. Central to this flexibility is the shift towards anabolism for allocation of central carbon surplus towards biosynthetic pathways generating an expanded need for electron acceptors in compartment specific manner [1, 2, 13, 14]. The pleiotropic mitochondrial role to utilize diverse carbon sources enabling cells to transition between different metabolic states positions mitochondria at the core of this metabolic flexibility [15].

Within the mitochondrial compartment metabolic alterations is majorly orchestrated by the Electron Transport Chain (ETC) accepting electrons from electron carriers through two points of entry- CI (Complex I, NADH dehydrogenase) and CII (Complex II, succinate dehydrogenase or SDH) and channel them to molecular oxygen [16–19]. CI, CIII and CIV organize into “respiratory supercomplexes” (SCs) for efficient transfer of electrons [20, 21]. CII exists independently and delivers electrons directly to CIII-CIV [22]. However, this concept has been debated with the proposal of fluid and plasticity models. The fluid model entails the respiratory units to be in random diffusion-collision mode to foster electron transfer [23], whereas the plasticity model reconciles both the concepts and proposes the MRCs to be in dynamic equilibrium between the free and supramolecular entities, allowing efficient optimization and adaptation of the electron flux [16, 24–26]. The manipulation of the electron flow across MRCs is influenced by several regulatory factors [27–30]. Particularly, in tumorigenesis, increased reliance on CI-driven OXPHOS activity has been associated with metastasis and chemoresistance [7, 31–35]. However, the set of regulatory factors that enable tumor cells to tailor mitochondrial electron flow, thereby achieving the metabolic plasticity necessary for sustained growth and metastasis, has been an active area of investigation.

Among the various regulators of MRCs, MCJ (DnaJC15) negatively regulates their functioning, and was first identified as a protein whose loss of expression promoted resistance of tumour cells to chemotherapeutic drugs [36–40]. Later, MCJ was identified as a negative regulator of CI [36]. Indirect increase in succinate levels by MCJ-loss promoted CI-respiration, acted as an epigenetic signal to increase the expression of MHC-I and antigen presentation genes; thereby, making the tumour cells more susceptible to CD8⁺ T-cell killing [20]. MCJ-deficient CD8⁺ T-cells also showed better tumour infiltration and anti-tumour activity, causing slower tumour growth and improved survivability [41]. MCJ is also considered as a potential target for pulmonary hypertension induced right ventricular failure since its absence led to protection of cardiac function under chronic hypoxia [42]. Loss of MCJ also has been associated with enhanced mitochondrial function in brown adipose tissue and UCP-1 independent/augmented thermogenesis, which protects against diet-induced obesity and fat accumulation [43].

In this study, we observed that higher MCJ levels was associated with increased cellular proliferative and migratory properties with complete dependence on mitochondrial respiration. MCJ shuttled mitochondrial respiration to CII-dependent bioenergetics by causing CI uncoupling and channeling of electrons through CII. This led to accumulation of NADH without compromising on ATP output, supporting lipogenesis and β-oxidation. CII-respiring cells were completely non-glycolytic and promoted aggressive primary tumour establishment. In summary, MCJ adapts the ETC to facilitate efficient balancing of ATP production, NAD+/NADH ratio, and coordinate the metabolic fluxes to meet the bioenergetic and biosynthetic demands of aggressive cell proliferation and migration.

## Results

### MCJ expression dictates cellular proliferative and migratory capacity

A pan-cancer analysis from TCGA (The Cancer Genome Atlas Program) datasets showed significant variability in the levels of MCJ in various cancer types and elevated expression of the protein in High Grade tumours (Fig. S1A, A’) which accounted for lower survivability (Fig. S1A”). Since, change in MCJ expression has been associated with disease-free survival and response to drugs in breast cancer [44], we modulated the expression of MCJ in breast cancer lines to check its association with growth and migration of the cells. Relative expression of MCJ under knock-out (*MCJ*^*KO*^*)* and overexpression (*MCJ*^*OE*^*)* condition were tested in different cells (MCF7, MDA-MB-231 and 4T1-Luc2) (Fig. S1B, C, E). The expression levels of two related genes, *DNAJC19* and *MAGMAS*, which are highly homologous to MCJ, did not show any alterations under *MCJ*^*KO*^ condition (Fig. S1B, C). To further validate the cellular phenotypes of *MCJ*^*KO*^ cells, *MCJ* was depleted in MDA-MB-231 cells using shRNA (Fig. S1D). The different cell lines were used to validate key findings across three breast cancer models (MCF7, MDA-MB-231 and the murine 4T1-Luc2 xenograft) which span different molecular subtypes and a in vivo context.

**Fig. 1:**
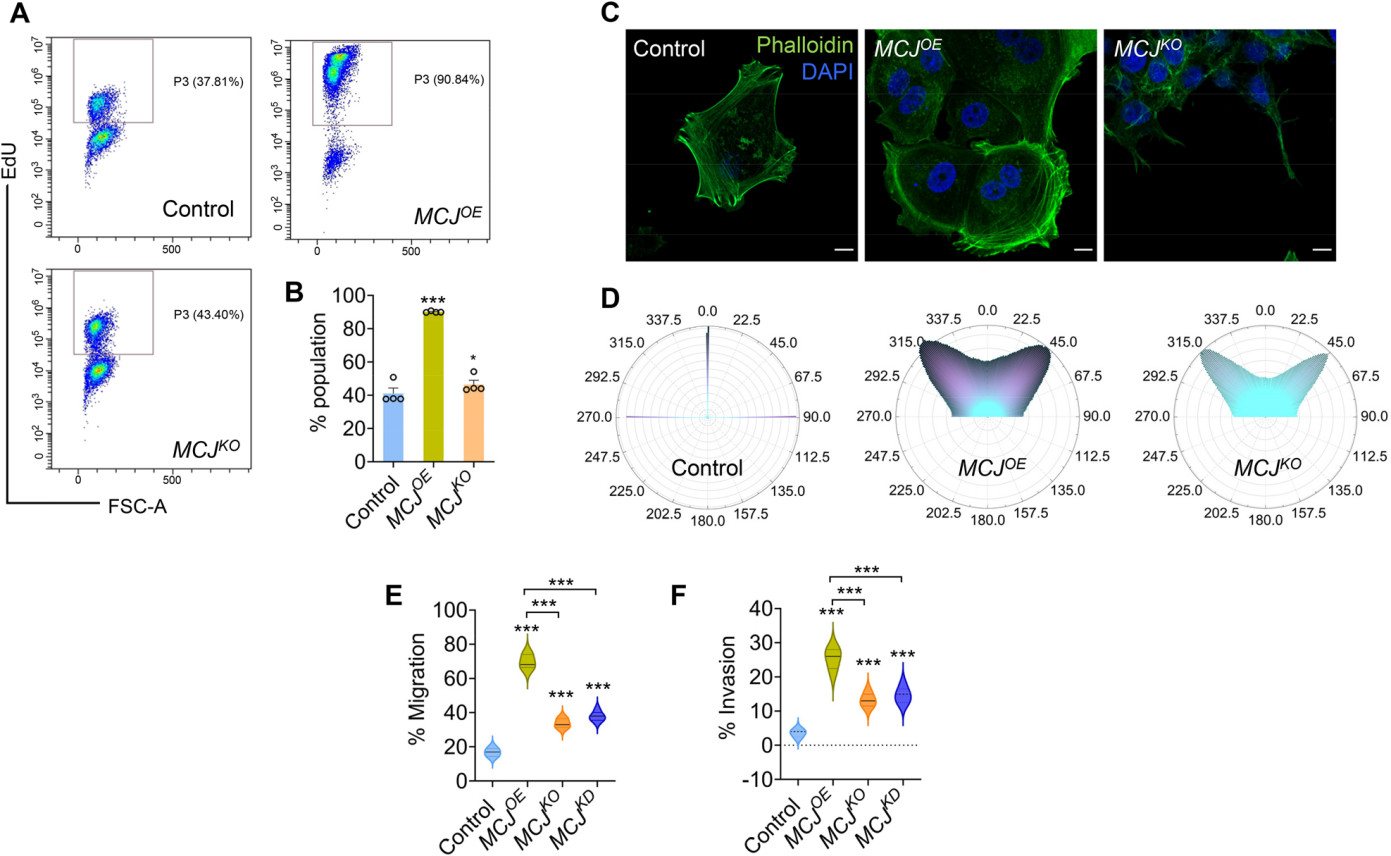
Enhanced MCJ level promotes aggressive tumourigenic phenotypes. **A**, **B** Flow cytometry contour plot depicting EdU^+^ cell population, further quantified in form of a graph, Bars denote mean ± s.e.m., *n* = 4, ^***^P(unpaired t-test)<0.0001 (**B**). **C**, **D** Phalloidin stained cells indicating reorientation of actin fibres, analyzed using Fiji to depict the changes in directionality and polarity (**D**). **E** The migrated cell populations across Boyden chamber with pore size 8 µm, using serum as a chemoattractant. Data points denote mean ± s.e.m., *n* = 5 experiments, ^***^P(unpaired t-test)<0.0001. **F** The invasive properties of the cell types were estimated through trans-well assay using Geltrex as extracellular matrix barrier. Data points refer mean ± s.e.m., *n* = 5 experiments, ^***^P(unpaired t-test)<0.0001. Statistical significance has been calculated compared to controls.

We found that *MCJ*^*OE*^ MCF7 cells incorporated higher amount of EdU in their DNA than *MCJ*^*KO*^, indicating their highly proliferative nature (Fig. 1A, B). The cells were also associated with changes in cellular morphology. *MCJ*^*OE*^ cells were notably larger with edge extensions compared to smaller filopodial projections of *MCJ*^*KO*^ (Fig. S1F). *MCJ*^*OE*^ cells displayed extensive lamellipodium network, resulting in a definitive leading-edge formation with cell body contraction and rear retraction (Fig. 1C, D), highlighting the property of migratory cells [45]. The morphological alterations correlated with robust migration rate of *MCJ*^*OE*^ over *MCJ*^*KO*^ cells and controls (Fig. 1E). Similarly, *MCJ*^*OE*^ cells possessed better capability to invade collagenous basement membrane (Fig. 1F) and showed higher instances of ~80 kDa cleaved E-cadherin extracellular domain (Fig. S1K) that is known to promote invasive phenotype by disrupting cell-cell adhesion and serving as anchoring points for migrating cells [46]. Similar results were observed in MDA-MB-231 cells where MCJ overexpressing cells showed morphological alterations (Fig. S1G, H) and were more migratory (Figs. S1I) and invasive (Fig. S1J) that the ones depleted for the protein.

### Differential proteomic remodeling reveals distinct cellular programs underlying MCJ alteration

**Fig. 2:**
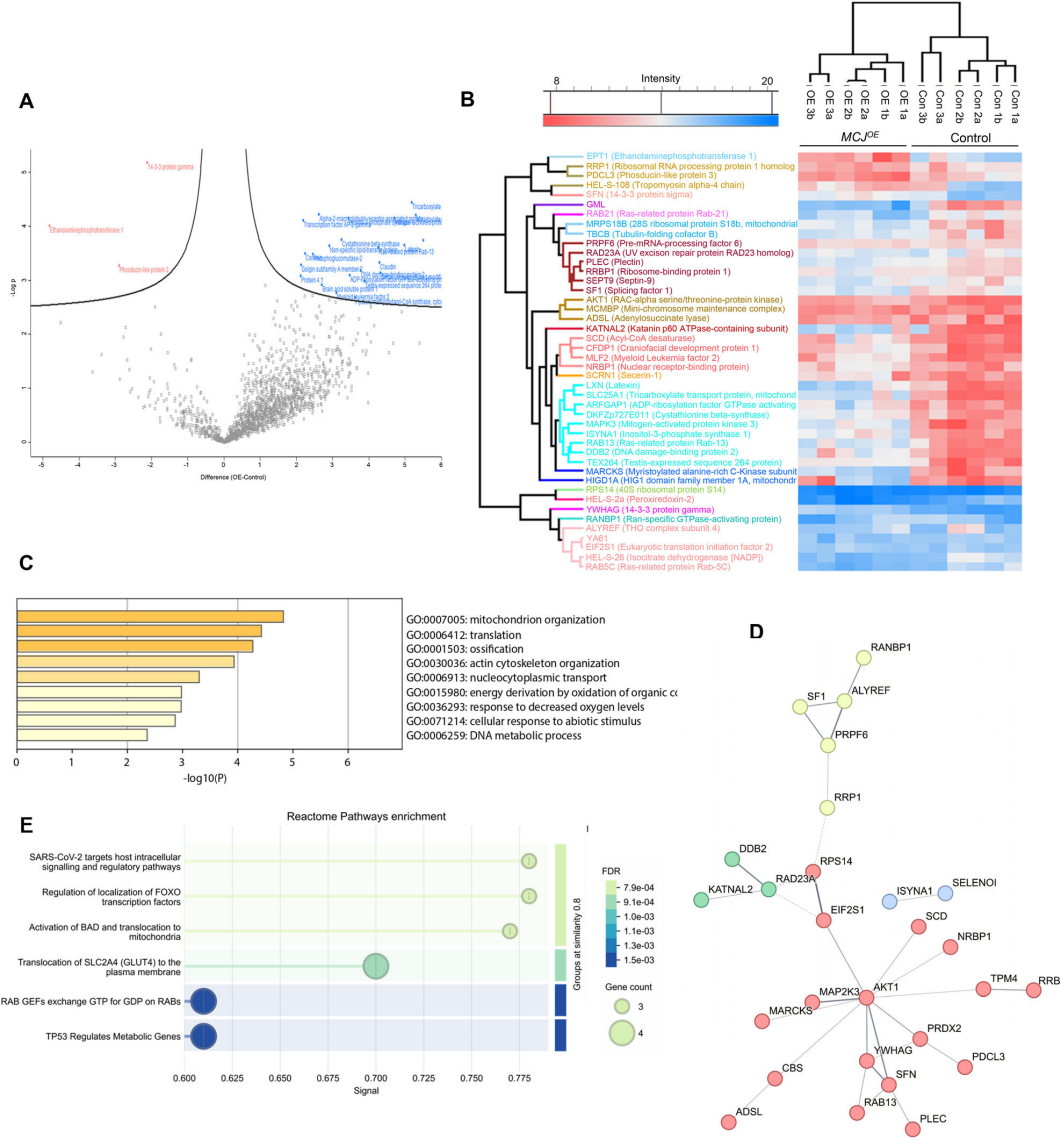
Proteomic analysis of MCJ overexpressing cells. **A** Volcanic plot (generated with Perseus) of the total upregulated and downregulated protein in OE background, having an FDR of 0.05 and s0:0.1. **B** Heat map plot (generated with Perseus) generated with ANNOVA significant proteins (with default significance threshold with *p*-value < 0.05) hierarchically clustered into 20 clusters representing the hierarchical clustering of proteins found to have significant differential expression between control and *MCJ*^*OE*^. **C** Enrichment bar graphs displaying GO biological processes with highest enrichment factor for control//OE calculated with Metascape (Fisher’s exact test) with ontology terms across different sources, such as GO, KEGG, and MSigDB, etc. **D** PPI network of Annova-significant proteins, segregated through k-means clustering, constructed using STRING v12.0 following the default settings. PPI enrichment p-value: 0.00985. **E** Functional enrichment of Reactome pathways grouped by similarity >=0.8 using STRING v12.0.

Proteomic profiling revealed a striking reorganization of the cellular protein landscape upon MCJ overexpression and reflected globally increased protein levels (Fig. 2A). Hierarchical clustering identified coherent modules of up- and downregulated proteins. In *MCJ*^*OE*^ cells, there was insignificant change in proteins associated with RNA processing but mitochondrial ribosome biogenesis factors (MRPS18B) and splicing factors were upregulated, indicating enhanced mitochondrial ribosomal assembly and spliceosomal activity. DNA repair factors such as *DDB2* were also elevated, indicating increased DNA damage recognition and genome surveillance capacity (Fig. 2B). Additionally, cytoskeletal regulators (*PLEC, MARCKS, KATNAL2, RAB13*) and signaling mediators (*MAP2K3, 14-3-3 proteins, SFN*) were enriched, consistent with remodeling of cytoskeletal dynamics and the proliferative and invasive nature of the cells (Fig. 2B). The metabolic and redox-related enzymes were also upregulated. These included *CBS* (cystathionine β-synthase), *ISYNA1* (inositol-3-phosphate synthase 1) and slight change in *ADSL* (adenylosuccinate lyase), suggesting reinforced amino acid, and phospholipid metabolism. The antioxidant enzyme *PRDX2* (peroxiredoxin-2) was also elevated, indicating strengthening of redox defenses (Fig. 2B). A Gene Ontology analysis of biological processes showed activation of processes associated with mitochondrial organization, cytoskeletal remodeling and carbon oxidation (Fig. 2C). Integration of the proteomic dataset with the STRING protein–protein interaction (PPI) network revealed highly connected clusters that mirror these functional themes. A major network centered on ribosomal proteins, RNA-processing factors, and translation regulators (Fig. 2D; *yellow nodes*). Another small cluster comprised the DNA repair and genome maintenance proteins, reflecting enhanced genome surveillance (Fig. 2D; *green nodes*). Notably, metabolic enzymes and redox regulators (such as CBS, ISYNA1, PRDX2, ADSL, SCD) also appeared within interconnected sub-networks, suggesting functional coupling of metabolic rewiring with stress adaptation mechanisms (Fig. 2D; *blue and red nodes*). Cytoskeletal and trafficking proteins were a part of Akt centered signaling and cytoskeletal module. AKT1 was present as the central hub, displaying extensive connectivity with proteins involved in cell survival and stress signaling pathways, including MAP2K3, MARCKS, PRDX2, and members of the 14-3-3 family (YWHA proteins, SFN) (Fig. 2D; *red nodes*). The idea was further supported by enrichment of reactome pathways that suggested sequestration of FOXO and Bad into the cytoplasm by Akt activated 14-3-3 proteins, leading to repression of pro-apoptotic genes and promoting cell cycle progression (Fig. 2E).

In contrast, *MCJ*^*KO*^ showed clustering of downregulated proteins (Fig. S2A, B) and coordinated downregulation of multiple metabolic and biosynthetic pathways within coherent functional modules (Fig. S2A). Gene Ontology (GO) analysis of biological processes revealed suppression of pathways associated with transcription, translation and vesicular transport (Fig. S2C). KEGG (Kyoto Encyclopedia of Genes and Genomes) pathway enrichment corroborated these observations, showing significant clustering of proteins involved in proteasome function, ribosome assembly, RNA metabolism and transport, all of which were predominantly reduced in the knockout condition (Fig. S2D). A limited subset of stress-responsive and cytoskeletal remodeling processes appeared relatively enriched, reflecting compensatory adaptations (Fig. S2B). A focused PPI network constructed using the top 50 ANOVA-significant proteins revealed four well-defined functional modules. The first two, centered on PABPN1 and PSMA1, encompassed proteins associated with mRNA processing, proteasomal degradation, and mitochondrial metabolism, which were collectively downregulated in MCJ knockout cells (Fig. S2E; *green and yellow nodes*). The third module, enriched for NOP14, WDR43, and EXOSC3, represented a ribosome biogenesis and RNA-processing hub, reflecting coordinated downregulation of nuclear and mitochondrial translational machinery (Fig. 2E; *red nodes*). In contrast, a smaller but distinct fourth cluster containing YAP1 and ROCK1, together with MYBBP1A linked mechanotransduction, cytoskeletal remodeling, and stress-responsive signaling with transcriptional regulation (Fig. S2E; *blue nodes*). This indicated that *MCJ*^*OE*^ and *MCJ*^*KO*^ might be promoting their respective enhancement of cell proliferative and invasive phenotypes, by following distinctive physiological programs.

### Atypical bioenergetic profiles define MCJ-modified cells

**Fig. 3:**
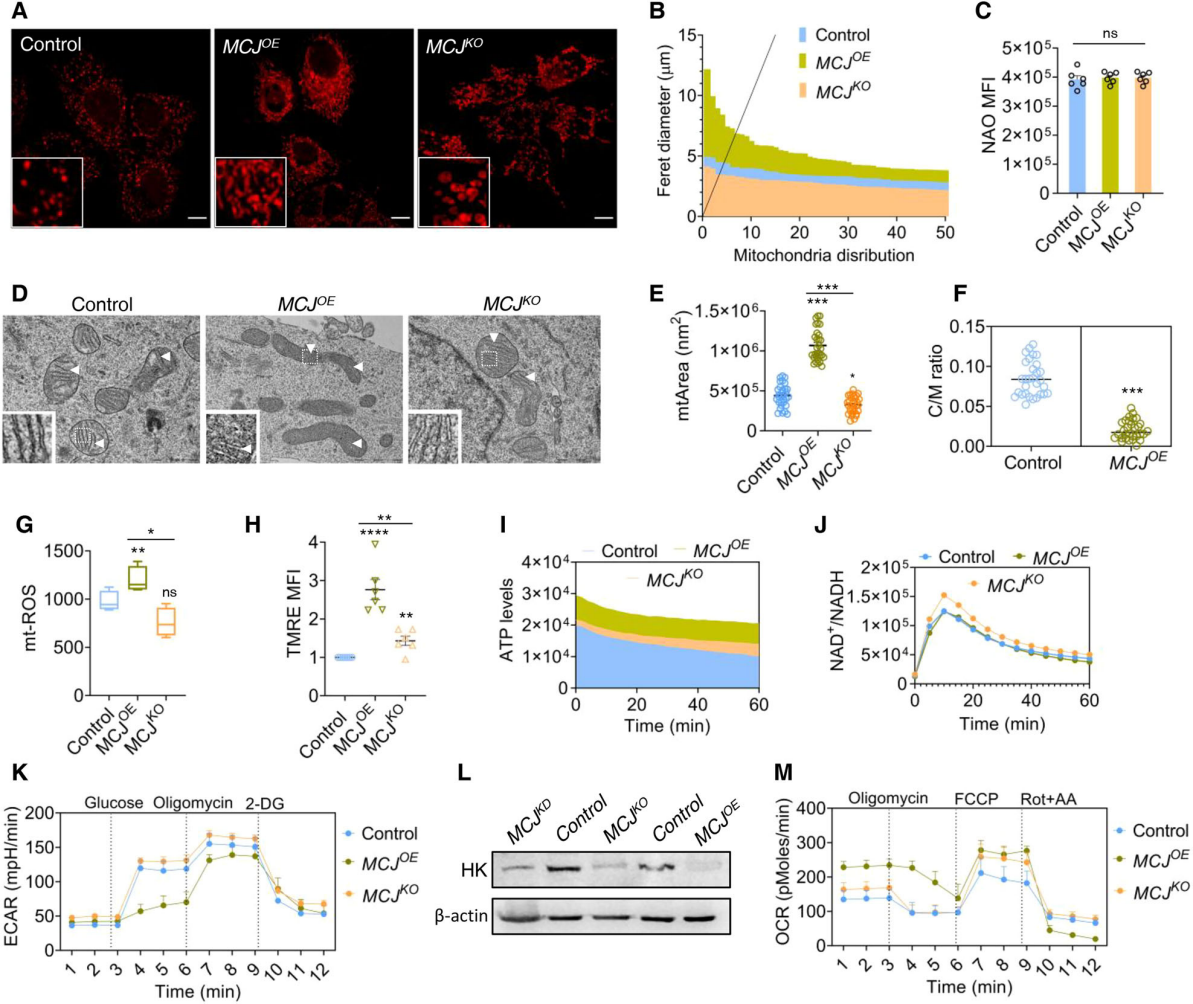
MCJ induces non-canonical structure-function alterations in mitochondria. **A**, **B** Representative confocal micrographs of cells overexpressing (*MCJ*^*OE*^) or deficient (*MCJ*^*KO*^) for MCJ stained with MitoTracker Red and analyzed using Fiji for their mean ferret diameter for 50 cells (**B**). Digitally zoomed image is shown as inset. Scale bar – 10 µm. **C** Mean fluorescence intensity (MFI) of cells stained with n-nonyl-acridine orange (NAO). Data represents mean ± s.e.m, *n* = 6, ns – not significant. **D** Electron micrograph of cell sections reflecting changes in mitochondria and cristae morphology. The cristae have been indicated by white arrowhead and also through digitally zoomed images as *inset*. **E** Quantification of the total mitochondrial area as in (**D**). Each data point represents mean mitochondrial size per cell, *n* = 30 cells, ^***^P(unpaired t-test)<0.0001, ^*^P(unpaired t-test)<0.01 compared to controls. **F** Cristae/mitochondrial area (C/M) ratio determined from (**D**). Each data point represents mean cristae area per mitochondria per cell, *n* = 30 cells, ^***^P(unpaired t-test)<0.0001. **G** Quantification of relative MitoSOX intensity in cells. Data represents mean ± s.e.m. *n* = 4 replicates. **H** Comparative retention of potential sensitive dye TMRE in the labelled cells, Data denotes mean ± s.e.m, *n* = 6 replicates. Area under the curve representation of the ATP (**I**) or NAD^+^/NADH ratio (**J**), *n* = 4 replicates^.^ *P(two-way Anova)<0.0001*. **K** ECAR measurement in cells with injection of 100 mM glucose, 1 mM Oligomycin and 2-DG injection, following starvation. Data shows mean values, *n* = 3 independent experiments. **L** Immunoblot against Hexokinase (HK) in cells. **M** Mean OCR levels in cells with injection of 1 μM Oligomycin, 0.5 μM FCCP and 1 μM Rotenone and Antimycin, *n* = 3 independent experiments. Each data point in the line plots for ECAR and OCR measurements represents mean with standard error for three independent experimental replicates, normalized per 10,000 cells (mpH/min/10,000 cells for ECAR and pMoles/min/10000 cells for OCR); *P(two-way annova)<0.0001* with respect to control. All experiments were performed in MCF7 cells. Scale bar – 10 microns. Uncropped blots are represented in Fig. S7.

The distinct phenotypic and molecular traits observed in *MCJ*^*OE*^ and *MCJ*^*KO*^ cells prompted us to investigate how MCJ, being a mitochondrial protein, influences the organelles’ structure and overall bioenergetic output. *MCJ*^*KO*^ MCF7 cells revealed presence of punctate mitochondria like control cells (Figs. 3A and S3A). In contrast *MCJ*^*OE*^ cells showed elongated fused mitochondrial morphology (Fig. 3A, B). There was no change in overall mitochondrial content or biogenesis as ascertained by consistent NAO (Figs. 3C and S3B) and PGC1a levels (Fig. S3C), respectively. Electron micrograph images of *MCJ*^*OE*^ cells showed presence of elongated mitochondria (Fig. 3D), covering substantially higher area (Fig. 3E), and possessing low cristae number/area (Fig. 3D; *inset* and F). In comparison, *MCJ*^*KO*^ mitochondria were more circular and had defined cristae morphology (Fig. 3D and 3D; *inset*).

*MCJ*^*OE*^ also generated the highest levels of superoxide (Fig. 3G), and contained hyperpolarized mitochondria (Figs. 3H and S3D). The variations in cristae shape and membrane polarity led us to test the energetics of cells. The ATP state of *MCJ*^*OE*^ cells were substantially higher than *MCJ*^*KO*^ and controls (Fig. 3I). But the redox ratio of NAD^+^/NADH was comparable for both *MCJ*^*OE*^ and control (Fig. 3J). *MCJ*^*KO*^ cells had elevated NAD^+^/NADH ratio, indicating higher utilization of these electron donors (Fig. 3J). Therefore, we predicted that elevated ATP levels in *MCJ*^*OE*^, could be due to upregulation of glycolysis. Glycolytic stress test showed reduced extracellular acidification rate (ECAR) with decreased glycolytic capacity and highest glycolytic reserve in *MCJ*^*OE*^ (Figs. 3K and S3E). Suppression of glycolysis in *MCJ*^*OE*^ was further supported by undetectable amounts of Hexokinase (Figs. 3L and S3F) and resistance of *MCJ*^*OE*^ ATP levels to glycolytic toxin 2-Deoxy-D-glucose (2-DG) (Fig. S3G). In contrast, both *MCJ*^*KO*^ and control cells showed presence of active glycolysis (Figs. 3K, L and S3E, F). The *MCJ*^*OE*^ had highest basal mitochondrial respiration resulting in elevated ATP production, least non-mitochondrial oxygen consumption and spare respiratory capacity, indicating that the higher ATP pool in these cells being completely mitochondrial (Figs. 3M, and S3H–J). The basal respiration, ATP production rate, and spare respiratory capacity for *MCJ*^*KO*^ was almost similar to controls (Fig. 3M, and S3H–J). The proton leak was highest in *MCJ*^*OE*^ cells, along with a lesser state III to state IV respiration (respiratory control ratio) for CI substrates, suggesting lower coupling efficiency in CI (Fig. S3K, L). This was further supported by reduced formation of I-III_2_-IV supercomplexes in case of *MCJ*^*OE*^ (Fig. S3M, N), as reported earlier [36]. The higher ATP state in-spite of a low RCR for CI indicated the possibility of bioenergetic rewiring in response to MCJ expression levels and differential channeling of electron flow under MCJ KO and OE conditions.

### MCJ adapts the preferential electron flow across the ETC complexes

**Fig. 4:**
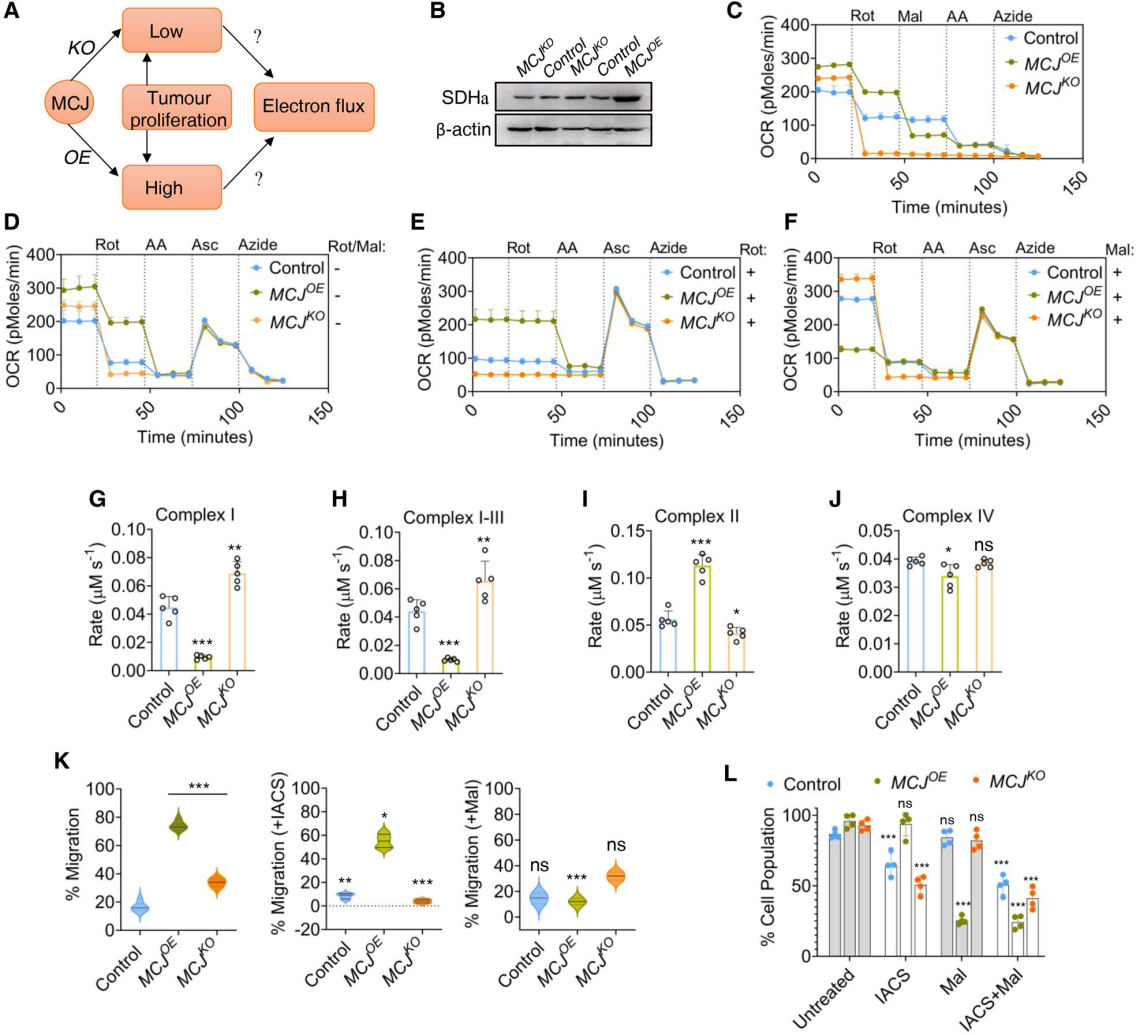
MCJ rewires the electron flow within ETC. **A** Schematic representation depicting association of MCJ overexpression (OE) or deficiency (KO) with tumour proliferative rates and electron flux. **B** MCF7 cells with altered expression of MCJ were immunoblotted with anti-SDHa antibody. **C** Determination of the flow of electrons across the respiratory complexes through subsequent injection of inhibitors for Complex I- Rotenone (Rot), Complex II- Malonate (Mal), Complex III- AA (Antimycin A), and Complex IV- Azide by electron flux assay. MCF7 cells were either left untreated (**D**) or pre-treated with Rotenone (**E**) or Malonate (**F**) prior to monitoring of electron flux after consecutive addition of complex inhibitors as mentioned above or addition of Ascorbate (Asc) as a substrate for Complex IV. Normalization of the OCR data has been performed per 10,000 cells (*y*-axis denote OCR as pMoles/min/10,000 cells). Each data point in the line plots represents mean with standard error for three independent experimental replicates; *P(two-way annova)<0.0001* with respect to control. Assessment of activities for Complex I (**G**), Complex II (**I**), Complex IV (**J**) and transfer kinetics of Complex I-III (**H**) in isolated mitochondria from MCF7 cells overexpressing MCJ or knocked-out for the protein. All enzyme activities are presented as the rate of reaction in µM/s normalized per 5 µg of protein. Bars denote mean ± s.e.m., *n* = 5 experiments, ^***^P(unpaired t-test)<0.0001, ^**^P(unpaired t-test)<0.001, ^*^P(unpaired t-test)<0.01. Statistical significance was calculated by comparing the activities of each genotype against control. **K** Migration of cell populations across Boyden chamber, using serum as a chemoattractant. The cells were either kept untreated (*left panel*), exposed to complex I inhibitor IACS-010759 (IACS) (*middle panel*) or complex II inhibitor malonate (Mal) (*right panel*). Data points denote mean ± s.e.m., *n* = 5 experiments, ^***^P(unpaired t-test)<0.0001, ^**^P(unpaired t-test)<0.001, ^*^P(unpaired t-test)<0.01. Statistical significance for untreated samples were calculated against the controls and for treated samples the corresponding untreated genotype was used as a reference for comparison. **L** Cell proliferative changes induced by Complex I inhibitor, IACS-010759 (IACS), Complex II inhibitor, Malonate (Mal) either alone or in combination, determined by MTT assay. Bars denote mean ± s.e.m., n = 8, ^***^P(one-way annova)<0.0001 compared against untreated samples of the respective genotypes.

We investigated whether the above observations were a consequence of MCJ-regulated electron flux through the MRCs (Fig. 4A). Alterations in MCJ did not cause any change in CI subunit, ND6 levels (Fig. S4A). *MCJ*^*OE*^ cells showed enhanced expression of succinic dehydrogenase a (SDHa) that accepts electrons from succinate into ETC, hinting towards its rewiring through CII (Fig. 4B). The electron flow in *MCJ*^*OE*^ was majorly blocked upon injection of Malonate, a CII inhibitor. Exposure of *MCJ*^*KO*^ cells to CI inhibitor Rotenone inhibited the electron flow, indicating electron entry through CI (Fig. 4C). A comparable reduction in the flux was observed upon repression of CIII by antimycin A and subsequent stimulation of CIV with an equivalent electron donor Ascorbate, suggesting a relatively uncompromised CIV activity, with the primary disparity being the preferential electron entry point (Fig. 4D). Hence, ETC rewiring by MCJ expression was further evaluated by pre-exposing the cells to either Rotenone or malonate. Rotenone pre-exposed *MCJ*^*KO*^ cells failed to respire, whereas *MCJ*^*OE*^ relatively remained unaffected (Fig. 4E). However, in the presence of malonate, *MCJ*^*OE*^ cells showed minimal basal respiration, and *MCJ*^*KO*^ ones got scantly affected. A slight reduction in electron flow was observed when *MCJ*^*OE*^ cells were treated with Rotenone (Fig. 4F), indicating a preferential electron flow through CII. *MCJ*^*OE*^ was associated with reduced CI activity (Fig. 4G), consistent with its reported role as an endogenous CI inhibitor [36]. The ensuing impairment of CI–CIII electron transfer kinetics (Fig. 4H) indicates a bottleneck in NADH-linked respiration, which likely imposed selective pressure for metabolic compensation. Indeed, we observed an upregulation of SDH expression (Fig. 4B) and enhanced CII activity in *MCJ*^*OE*^ mitochondria (Fig. 4I), suggesting a metabolic shift toward succinate oxidation to sustain electron flux into the respiratory chain. *MCJ*^*KO*^ mitochondria expectedly possessed higher CI activity and CI-CIII transfer kinetics than controls (Fig. 4G, H). Notably, although not resolved in electron flux assays, direct measurement of individual CIV activity indicated a minor reduction under *MCJ*^*OE*^ conditions compared to controls (Fig. 4J). Along with CI uncoupling, this reduced capacity to transfer electrons to oxygen could account for the elevated ROS levels observed in *MCJ*^*OE*^ cells (Fig. 3G). The terminal step of oxidative phosphorylation appeared unaffected in the *MCJ*^*KO*^ background (Fig. 4J). Comparable alterations in respiratory complex activities were also observed upon modulation of MCJ expression in MDA-MB-231 cells (Fig. S4B), along with changes in expression levels of SDH and Hexokinase (Fig. S4C), indicating elevated CII activity and suppressed glycolysis.

The electron flow data were further validated by sensitivity of the MCJ-altered cells to toxins of the respiratory complexes. In line with their reduced dependence on CI respiration, *MCJ*^*OE*^ cells were resistant to Complex I inhibitor IACS-010759 with minimal changes in proliferative and migratory phenotypes but were sensitive to Complex II inhibitor malonate (Fig. 4K, L). In contrast, *MCJ*^*KO*^ showed highest sensitivity to IACS-010759 and was relatively immune to malonate. Co-treatment with both IACS-010759 and malonate completely inhibited *MCJ*^*OE*^ proliferation (Fig. 4L), indicating its respiratory dependence to be mitochondrial. Knockdown of MCJ in MDA-MB-231 cells showed a similar change in the proliferative rates when treated with respective Complex I and Complex II inhibitors (Fig. S4D), where *MCJ*^*OE*^ cells were insensitive to IACS-010759 and showed reduced growth in presence of malonate. In summary, results indicated that electron flow in *MCJ*^*KO*^ were primarily through CI and for *MCJ*^*OE*^, the III_2_-IV complexes were fed primarily by electrons entering through CII.

### MCJ induced CII-flux over CI promoted anabolic and catabolic metabolism of lipids

The suppression of glycolysis in non-canonical Complex II-dependent electron channeling under *MCJ*^*OE*^ condition, persuaded us to investigate the preferential substrates for the rewired MRC electron flow driving mitochondrial oxidation.

**Fig. 5:**
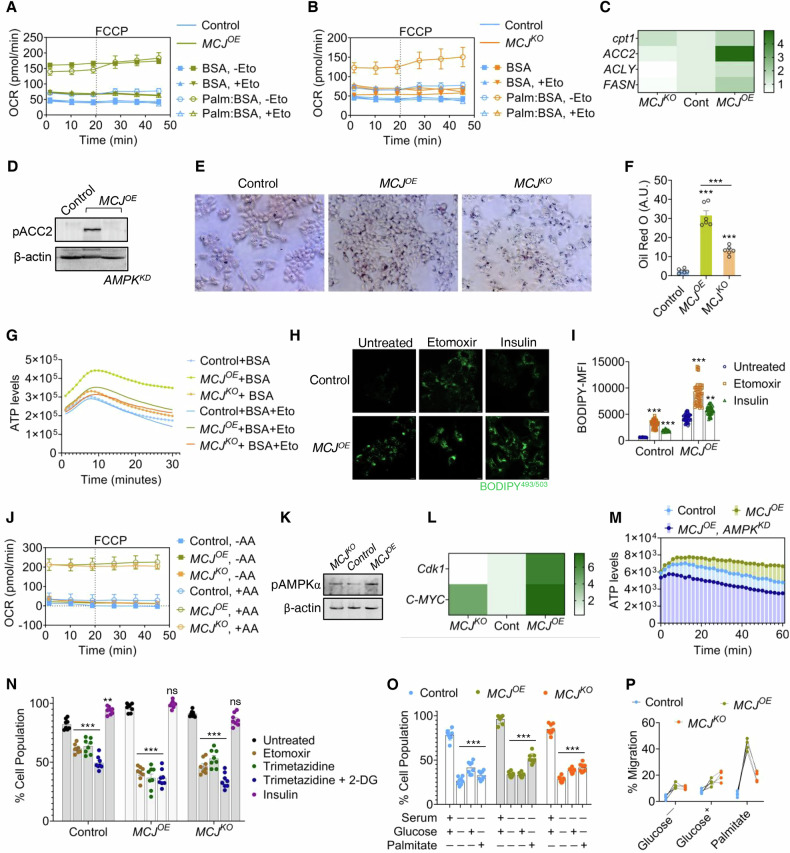
CII bioenergetics reprograms metabolism to fatty acid utilization and synthesis. Mean Mitochondrial OCR values, in cells overexpressing (**A**) or impaired (**B**) for MCJ, representing the fatty acid oxidation after addition of Palmitate (Palm) conjugate/only BSA/ Palmitate conjugate ± Etomoxir (Eto) to the cells, following starvation for 6 h. Only one FCCP injection was administered, *n* = 3 independent replicates, *P(two-way annova)<0.0001* with respect to control. The assay was performed simultaneously allowing comparison between (**A**) and (**B**), and data was normalized per 10,000 cells (OCR as pMoles/min/10,000 cells). **C** Heat map depicting the fold transcript levels of the genes associated with fatty acid metabolism. Data represents the mean intensity profile of 3 replicates; *P(two-way Anova)<0.0001* with respect to control. **D** Immunoblot representing levels of pACC2 in *MCJ*^*OE*^ cells with or without AMPK depletion. **E**, **F** Estimation of the lipid droplets accumulation in the cells were made by staining with Oil Red O, following by its quantification. Bars denote mean ± s.e.m, *n* = 6, ^***^P(unpaired t-test)<0.0001, ^**^P(unpaired t-test)<0.001. **G** Area under the curve representing the mean ATP levels in cells with altered MCJ levels treated or untreated with Etomoxir (Eto), when supplemented with BSA only; *n* = 4, *P(two-way Anova)<0.0001* with respect to control. **H**, **I** Representative images of lipid accumulation in MCJ overexpressing and control cells upon Etomoxir treatment, visualized by fluorescence imaging of BODIPY stain. Insulin was used as an internal control. Mean fluorescence intensity (MFI) of BODIPY staining determined through 30 independent fields of view (**I**). Bars represent mean ± s.e.m, *n* = 30, ^***^P(unpaired t-test)<0.0001. **J** Mean Mitochondrial OCR values for cells left untreated or spiked with amino-acid (AA) cocktail, *n* = 3 independent replicates; *P(two-way Anova)<0.0001* with respect to control, *y*-axis denote OCR as pMoles/min/10,000 cells. **K** Blot immunolabelled with anti-AMPK antibody reflecting levels of the protein. **L** Heat map depicting the fold transcript levels of cMyc and Cdk1. Data represents the mean intensity profile of 3 replicates; *P(two-way Anova)<0.0001* with respect to control. **M** Area under the curve representing the mean ATP in MCJ expressing cells depleted for AMPK, *n* = 4, *P(two-way Anova)<0.0001*. **N** Equal number of cells were treated with fatty acid oxidation inhibitors Etomoxir, Trimetazidine with or without glycolytic toxin 2-DG and their ability to proliferate was measured through MTT assay. Insulin that promotes lipid storage was used as an internal control. Bars denote mean ± s.e.m., *n* = 8, ^***^P(unpaired t-test)<0.0001, ^**^P(unpaired t-test)<0.001 compared to untreated control genotype. **O** The ability of the cells to proliferate in minimal media supplemented with either glucose or palmitate, determined through MTT assay. Media enriched with serum and glucose served as control and was used to calculate statistical significance. Bars denote mean ± s.e.m., *n* = 8, ^***^P(unpaired t-test)<0.0001. **P** The migrated cell population, growing in minimal media supplemented with either glucose or palmitate, across Boyden chamber using serum as chemoattractant. Data points denote *n* = 4 experiments, ^***^P(two-way annova)<0.001. All experiments were performed in MCF7 cells. In (**N**) and (**O**), data was normalized against sample with maximum absorbance value which was set to 100%. Statistical significance for the treated was calculated with respect to untreated of corresponding genotype, *ns – not significant*. All experiments were performed in MCF7 cells. Uncropped blots are represented in Fig. S7.

*MCJ*^*OE*^ cells featured highest OCR values, indicating oxidation of FAs (Fig. 5A), which got impeded upon addition of Etomoxir, an inhibitor of carnitine palmitoyltransferase-1 (CPT1). CPT-1 catalyzes conversion of FA catabolite acyl-CoA to acylcarnitine that is transported into mitochondria to generate acetyl-CoA and NADH [47]. *MCJ*^*KO*^ cells were partially dependent on oxidation of palmitate (Fig. 5B), and the rate of FA oxidation was less than the *MCJ*^*OE*^ cells (Fig. S5A). *MCJ*^*OE*^ cells did show an elevation in CPT-1 transcript and protein levels, along with high ACC2 transcripts and pACC2 that serve as a marker for FAO (Figs. 5C, D and S5C). *MCJ*^*OE*^ cells surprisingly showed high OCR levels in experimental control BSA (Fig. 5A). The difference, however, was insignificant relative to palmitate:BSA conjugate (*P* = 0.3920). The spare respiratory capacity of Palmitate injection was better than BSA only controls (Fig. S5D). *MCJ*^*OE*^ cells showed higher lipid reserves (Fig. 5E, F) compared to *MCJ*^*KO*^ and control cells, which possibly contributed to elevated basal OCR observed under experimental control BSA (Fig. S5B). The higher basal OCR of *MCJ*^*OE*^ in presence of BSA, was considerably reduced upon Etomoxir injection (Fig. S5B), along with lowering of ATP levels (Fig. 5G), indicating sustenance of higher ATP levels in *MCJ*^*OE*^ was through active FAO. Similar reduction in ATP levels was found when *MCJ*^*OE*^ cells were exposed to Trimetazidine, an inhibitor of 3-ketoacyl-CoA thiolase (Fig. S5E), an enzyme associated with FA metabolism. Etomoxir treatment also resulted in comparatively higher accumulation of lipid droplets in *MCJ*^*OE*^ cells than controls (Fig. 5H, I). Insulin, known to promote FA synthesis, was used as an internal control. Palmitate in lipid droplets is generated from citrate through combined activities of ATP citrate lyase (ACLY), ACC2 and FASN [47]. The expression levels of these genes were upregulated in *MCJ*^*OE*^ cells (Fig. 5C). *MCJ*^*OE*^ also showed increased levels of IDH1 (Fig. S5F), that generates citrate through reductive carboxylation of glutamine derived α-ketoglutarate, indicating possible existence of enhanced citrate flux towards fatty acid synthesis via ACLY [47, 48]. The observation was supported by proteome analysis that displayed higher levels of IDH and mitochondrial-cytoplasmic citrate carrier SLC25A1 (Fig. 2B). Indeed, supplementation of amino acids resulted in their higher utilization as substrates for mitochondrial respiration in *MCJ*^*OE*^ cells (Fig. 5J). Together with the elevated α-ketoglutarate/citrate ratio (Fig. S5G), this suggests an increased flux through the reductive carboxylation pathway of glutamine to citrate, supporting lipid biosynthesis. However, we did not observe a consequent change in mTOR (Fig. S5H), instead *MCJ*^*OE*^ showed increased pAMPK (Figs. 5K and S5I) and cMyc along with its downstream effector Cdk1 (Fig. 5L). The higher levels of AMPK substrates pACC2 and Cpt1 in *MCJ*^*OE*^, were subsequently reduced upon AMPK depletion (Figs. 5D and S5C), indicating an active FAO promoted by AMPK. Knock-down of AMPK lowered the elevated ATP pool in *MCJ*^*OE*^ (Fig. 5M). The results demonstrate that rewiring of the preferential electron flow through CII led to consequent shift from glycolysis (Fig. 3K, L) and being majorly sustained by amino acid metabolism, lipogenesis, and activation of fatty acid oxidation (FAO). The cellular metabolic dependency was further examined by monitoring growth upon treatment with metabolic inhibitors. The growth of *MCJ*^*OE*^ cells was lowest upon treatment with FAO toxins Etomoxir and Trimetazidine when compared to control or *MCJ*^*KO*^ cells (Fig. 5N). The growth of both control and *MCJ*^*KO*^ slightly slowed under FAO toxins but further reduction in their survivability was seen under Trimetazidine:2-DG co-treatment, indicating co-dependence of these cells to both FA metabolism and glycolysis (Fig. 5N). In comparison, no incremental change was observed in MCJ overexpressing cells when co-treated with Trimetazidine:2-DG (Fig. 5N). Expectedly, *MCJ*^*OE*^ population expanded more in palmitate influxed minimal media and were unresponsive to glucose supplementation, whereas *MCJ*^*KO*^ showed incremental changes to glucose or palmitate enriched media (Fig. 5O). Parallel changes were observed in the migratory ability MCJ overexpressing or knock-out cells under different media supplementations (Fig. 5P). Together, these observations indicate dependence of CII-respiring *MCJ*^*OE*^ cells to lipid metabolism. The results were further validated in MDA-MB-231 cells where *MCJ*^*OE*^ cells showed accumulation of lipid droplets and poorer growth in presence of FAO inhibitors (Fig. S5J–M).

### CII-dependent absolute mitochondrial respiration concord with primary tumour burden

**Fig. 6:**
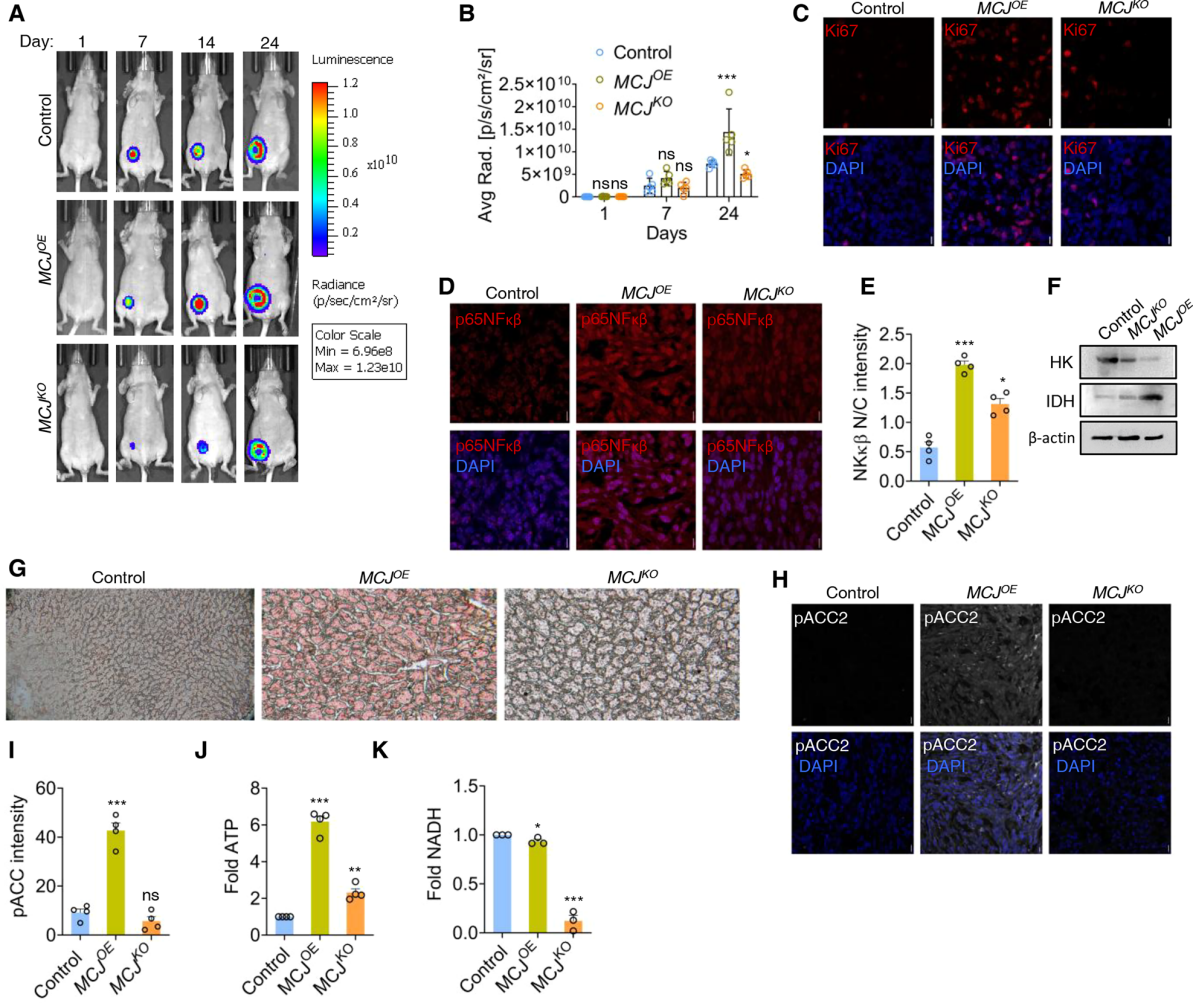
MCJ induced CII-respiration results in increased primary tumour. **A** Representative in vivo bioluminescence imaging of primary tumour generated from 4T1Luc2, cells in orthotopic control, *MCJ*^*OE*^ and *MCJ*^*KO*^ at different time intervals after cell implantation. The colour scale indicate average radiance (photons/sec/cm^2^/super pixel) emitted. **B** Tumour growth curve representing temporal change in the average radiance emitted from the region of interest in each group. Data points represent mean of *n* = 5 mice in each group, ^***^P(unpaired t-test)<0.0001, ^*^P(unpaired t-test)<0.01 calculated by comparing the individual genotypes with the control of corresponding time-point, ns – non-significant. **C** Confocal micrographs of cryosections derived from primary tumours were immunodecorated by anti-Ki67 antibody. **D**, **E** Confocal microscopy images of anti-p65NFκβ immunolabeled samples. The mean nuclear to cytoplasmic (N/C) distribution of p65NFκβ (E) was represented as bar graph, *n* = 4. **F** Immunoblot assay from primary tumour lysate using anti-Hexokinase (HK), and Isocitrate Dehydrogenase (IDH). **G** Oil O red staining (pink colour) of secondary site tumour section in liver dissected from each group. Scale bar – 100 pixels. **H**, **I** Confocal microscopy images of anti-pACC2 immunolabeled samples. The pACC2 labeling intensity was represented as bar graph, *n* = 4. Fold change in the ATP (**J**) and NADH (**K**) levels in the primary tumour lysates of each group, *n* = 4 mice from each group. For quantification graphs of the confocal images, each data point represents mean of 5 image slices from each group. ^***^P < *0.0001,* ^**^P < *0.001,* ^*^P < *0.01* compared to control*, ns -non-significant for unpaired t-test*. Scale bar – 10 microns. Uncropped blots are represented in Fig. S7.

*MCJ*^*OE*^, *MCJ*^*KO*^ or control 4T1-Luc2 isogenic breast cancer lines were implanted in immunodeficient mice, and the primary tumor burden was determined by luciferase activity. *MCJ*^*KO*^ mice, following robust CI-dependent respiration, showed minimal changes in tumor load compared to controls (Fig. 6A, B). In contrast, CII-respiring *MCJ*^*OE*^ mice formed comparatively large sized primary tumours (Fig. 6A) and their growth rate was consistently higher than *MCJ*^*KO*^ mice and controls (Fig. 6B). *MCJ*^*OE*^ showed higher number of Ki-67 foci (Figs. 6C and S6A), indicating enhanced proliferative rates. The hematoxylin-eosin staining of frozen tumour sections indicated existence of collective cell migration pattern (Fig. S6B). The proliferative zone of *MCJ*^*OE*^ mice showed elevated p65NF-κβ expression and nuclear translocation (Fig. 6D, E). The mice results were recapitulated by a meta-analysis of TCGA Breast Cancer database, which showed that the primary tumour cohort was associated with increased MCJ expression (Fig. S6C) and lower survivability (Fig. S1A). We hypothesized from our previous cell-based observations that *MCJ*^*OE*^ promoted the aggressive tumour growth due to non-glycolytic mitochondrial respiration. The undetectability of Hexokinase II in *MCJ*^*OE*^ mice supported the notion that maintenance of cellular energy demand is primarily mitochondrial (Fig. 6F), sustained from the accumulated lipid reserves as observed in *MCJ*^*OE*^ mice where considerable cytoplasmic area was covered with lipid droplets in both primary and secondary sites (Figs. 6G and S6D). The activation of FAO was corroborated with higher levels of IDH1 and pACC2 labelling in *MCJ*^*OE*^ tissues (Fig. 6F, H, I). The lipid metabolism driven respiration resulted in elevated ATP but unchanged NADH levels in *MCJ*^*OE*^ tissues, indicating non-involvement of NADH oxidation for ATP generation (Fig. 6J, K). In contrast, *MCJ*^*KO*^ tissues, following CI-dependent respiration showed substantial reduction in NADH levels (Fig. 6J, K). In summary, the 4T-Luc2 xenografts further validated the proliferative and metabolic data obtained from cultured cells.

## Discussion

Metabolic plasticity represents a defining hallmark of tumor progression, allowing cancer cells to rapidly adapt to fluctuating nutrient availability and energetic demands. This adaptation supports their elevated energy demands manifested in their intensive morphological and cytoskeletal changes associated with aggressive proliferative and migratory behaviours [45, 49]. Our study identifies the mitochondrial protein MCJ as a central determinant of this adaptive response. MCJ is known to associate with Complex I (CI) of the mitochondrial respiratory chain, functioning as an endogenous negative regulator of CI activity [36, 50]. Overexpression of MCJ inhibited CI-linked respiration and enhanced apparent reliance on Complex II (CII)-supported oxidative metabolism, whereas MCJ knock-out produced an opposing effect. This bidirectional control underscores MCJ’s role as a rheostat that tunes electron entry into the ETC. By rerouting electron flow from Complex I (CI) to Complex II (CII), MCJ establishes a metabolic state characterized by reliance on FAO, suppressed glycolysis, and sustained oxidative metabolism. This reorganization not only supports the energy-intensive requirements of proliferation, migration, and cytoskeletal remodeling but also reveals a metabolic vulnerability in tumors.

Classically, CI has been considered the principal entry point for mitochondrial respiration. Mutations and perturbation of CI diverts the carbon feed into the TCA cycle away from glycolysis to glutamine [51]. MCJ^OE^ exhibited signatures of CI deficiency coupled to enhanced CII substrate utilization. In addition to CI, CII substrates and mutations have been associated with enhanced grades of tumour [52, 53]. CI dysfunction perturbs electron carrier regeneration and stalls TCA cycle, thereby influencing purine transport and one-carbon metabolism, that are intermittently connected with cellular NAD+/NADH levels [54–56]. Shunting the flow of electrons through CII ensures the supply-demand of these electron carriers for anabolic processes and stress responses, without compromising on the overall energy output from MRCs. Since, CI-respiration is partly glycolytic with enhanced feed into TCA cycle, induction of catabolic mechanisms such as FAO is primarily aimed at supplementing energy production [36]. Under MCJ promoted CII-respiration, lactate generation was inhibited due to suppressed glycolysis, causing CII to follow alterative bioenergetics processes. The rewired ETC electron flow through CII sustained an increased ATP yield and de novo lipogenesis probably, aided by the availability of the electron donors [57]. In absence of glycolysis, glutamine was utilized to generate lipid surplus for their subsequent oxidation. The anabolic fixation of carbon was observed in spite of AMPK being activated and unaltered levels mTORC1 or PGC1α [58]. Conventionally, AMPK induced under energetic stress conditions, possesses a tumour suppressive role by acting as a metabolic checkpoint that restrains cell proliferation, and its inactivation channels the cells to aerobic glycolysis [59]. In our case, AMPK acts here as a contextual oncogene, enforcing a pro-survival program essential for overcoming the specific metabolic stress induced by CI impairment and suppressed glycolysis. AMPK maintained higher ATP levels in *MCJ*^*OE*^ via promotion of FAO through phosphorylation of ACC2. This metabolic flux is critical for generation of reducing equivalents to manage the severe oxidative stress [60]. Knockdown of AMPK resulted in decreased cellular ATP levels, demonstrating its role in sustaining MCJ-driven bioenergetics. The co-upregulation of oncogenic transcription factors such as Myc which promotes glutamine metabolism and maintains respiratory capacity [61], may possibly underlie this shift and might be providing the necessary signal override that exploits AMPK’s pro-survival functions while bypassing its anti-proliferative signaling pathways [61]. Although CI-respiration is also augmented by FAO [43, 62], complete reliance on FAO under MCJ-driven CII respiration exposes a metabolic liability. While this rewiring supports tumor growth and survival, it also renders cells hypersensitive to pharmacological inhibitors of fatty acid metabolism. Indeed, CII-dependent tumors may be uniquely susceptible to dual blockade of FAO and glutamine utilization. Moreover, mitochondrial DNA mutations compromising CI and elevating succinate oxidation have been linked to aggressive prostate cancers, supporting the broader relevance of this mechanism [52]. Thus, targeting MCJ-mediated metabolic flexibility represents a promising strategy to suppress both primary tumor growth and acquisition of invasive phenotypes.

Collectively, these findings underscore the complexity of mitochondrial adaptations in cancer, and this study provides a perspective on how a tumour might switch its bioenergetic profile as per its demands and liabilities. Future research should aim to dissect the mechanistic basis by which MCJ disengages CI and preferentially drives CII utilization, including possible regulation through post-translational modifications and oncogenic transcriptional programs. A second critical avenue is to map in detail how this rewiring redistributes carbon flux across glycolysis, glutamine metabolism, and lipid biosynthesis, and how such redistribution contributes to mitochondrial morphological adaptations, tumor plasticity and role of AMPK under MCJ-driven respiration.

In conclusion, our work reveals MCJ as a key modulator of mitochondrial bioenergetic plasticity, orchestrating a switch from CI- to CII-dependent respiration that sustains tumor growth yet uncovers exploitable metabolic liabilities. Recognizing this plasticity not as a static trait but as a dynamic, stage-specific adaptation provides new opportunities for therapeutic intervention, particularly through precision targeting of FAO and CII metabolism in MCJ-driven tumors.

## Supplementary information

Supplementary Figure S1-S7

Supplemental Methods

## Data Availability

The data that supports the findings of this study are available within the main text and its Supplementary Information file. The lead contact will share all raw data associated with this paper upon reasonable request and, when applicable, fulfilment of appropriate material transfer agreements. The raw files of Proteomic datasets is available at ftp://MSV000096213@massive.ucsd.edu.
