# Supplementary material for: MCJ modulates mitochondrial ETC flux to promote lipid metabolism–driven enhancement of cell proliferation and migration
Source: Cell Death Dis. 2026 Jan 2;17(1):151. doi: 10.1038/s41419-025-08398-y (PMC12859062; doi:10.1038/s41419-025-08398-y)
Supplement: Supplementary file 1 — Supplementary Figure S1-S7 [file 41419_2025_8398_MOESM1_ESM.pdf]

Figure S1

A

MCJ expression in different Tumour types

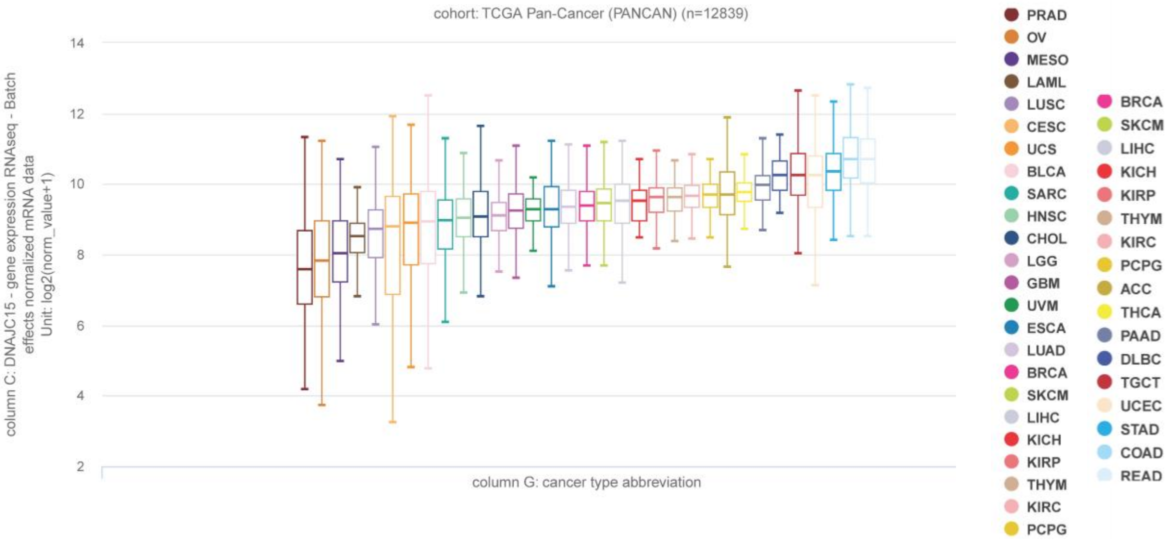

A'

MCJ expression in different Tumour grades

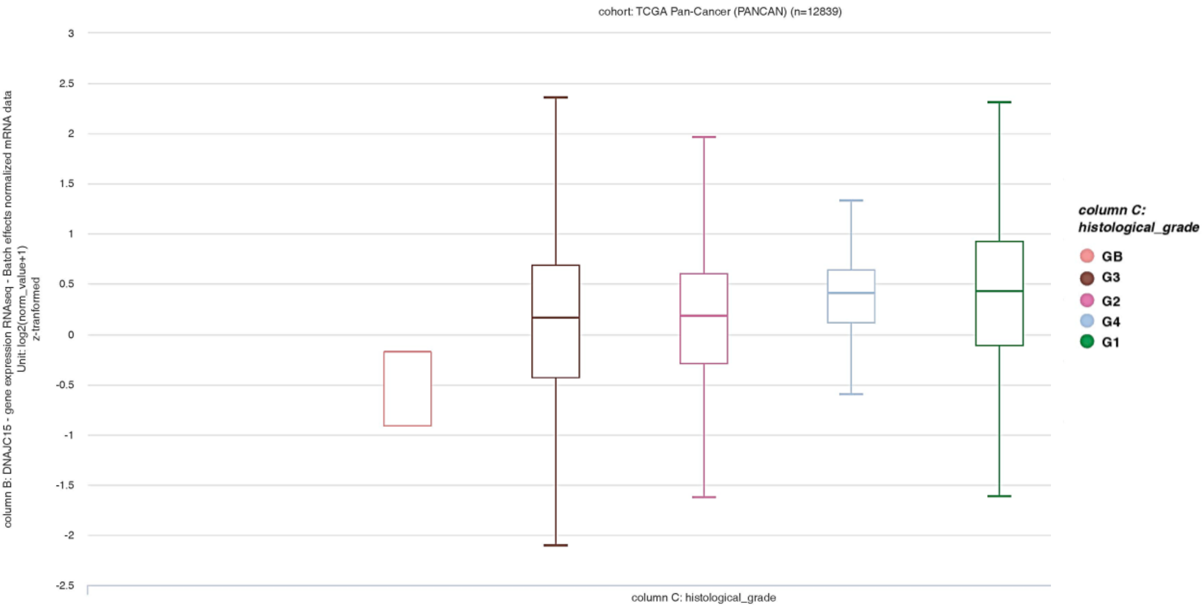

A''

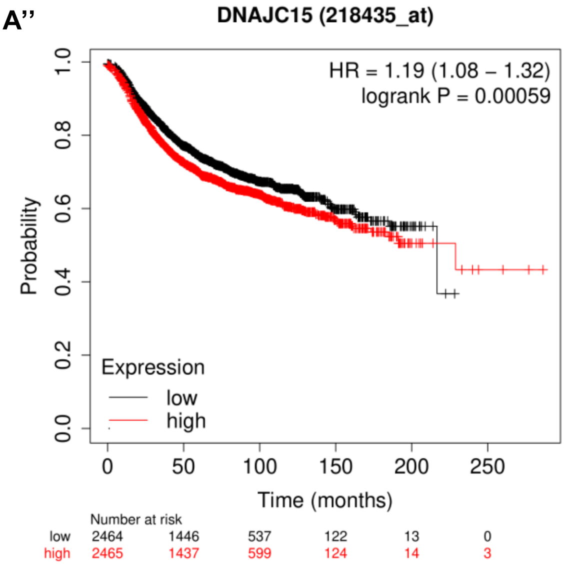

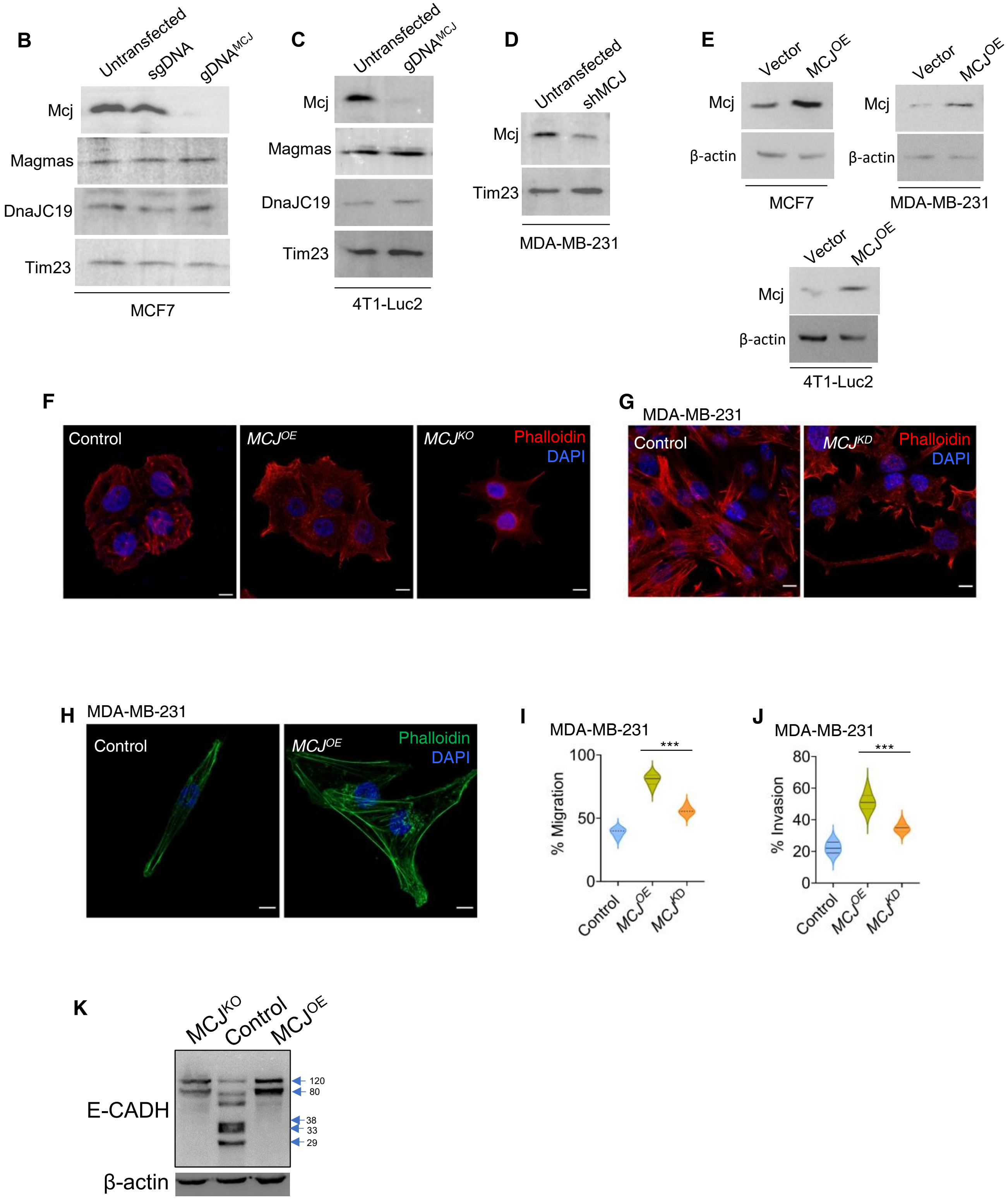
B
Untransfected
sgDNA
gDNA^MCJ
Mcj
Magmas
DnaJC19
Tim23
MCF7
C
Untransfected
gDNA^MCJ
Mcj
Magmas
DnaJC19
Tim23
4T1-Luc2
D
Untransfected
shMCJ
Mcj
Tim23
MDA-MB-231
E
Vector
MCJ^OE
Mcj
β-actin
MCF7
Vector
MCJ^OE
Mcj
β-actin
MDA-MB-231
Vector
MCJ^OE
Mcj
β-actin
4T1-Luc2
F
Control
MCJ^OE
MCJ^KO
Phalloidin
DAPI
G
MDA-MB-231
Control
MCJ^KD
Phalloidin
DAPI
H
MDA-MB-231
Control
MCJ^OE
Phalloidin
DAPI
I
MDA-MB-231
***
% Migration
100
50
0
Control
MCJ^OE
MCJ^KD
J
MDA-MB-231
***
% Invasion
80
60
40
20
0
Control
MCJ^OE
MCJ^KD
K
MCJ^KO
Control
MCJ^OE
E-CADH
120
80
38
33
29
β-actin

Figure. S1. (**A, A', A''**) MCJ expression is associated with tumourigenesis and poor survivability. All analysis has been performed using TGCA Pancan database. Expression levels of MCJ (DNAJC15) in multiple cancer types (A) or tumour histological grades (A'), derived from The Cancer Genome Atlas (TCGA) pancancer dataset. The cancer types include Prostate Adenocarcinoma (PRAD), Ovarian Serous Cystadenocarcinoma (OV), Mesothelioma (MESO), Acute Myeloid Leukemia (LAML), Lung Squamous Cell Carcinoma (LUSC), Cervical Squamous Cell Carcinoma and Endocervical Adenocarcinoma (CESC), Uterine Carcinosarcoma (UCS), Bladder urothelial carcinoma (BLCA), Sarcoma (SARC), Head and Neck Squamous Cell Carcinoma (HNSC), Cholangiocarcinoma (CHOL), Brain Lower Grade Glioma (LGG), Glioblastoma Multiforme (GBM), Uveal Melanoma (UVM), Esophageal Carcinoma (ESCA), Lung Adenocarcinoma (LUAD), Breast Invasive Carcinoma (BRCA), Skin Cutaneous Melanoma (SKCM), Liver Hepatocellular Carcinoma (LIHC), Kidney Chromophobe (KICH), Kidney Renal Papillary Cell Carcinoma (KIRP), Thymoma (THYM), Kidney Renal Clear Cell Carcinoma (KIRC), Pheochromocytoma and Paraganglioma (PCPG), Adrenocortical Carcinoma (ACC), Thyroid Carcinoma (THCA), Pancreatic Adenocarcinoma (PAAD), Diffuse Large B-Cell Lymphoma (DLBC), Intratubular Germ Cell Tumor (ITGCT), Uterine Corpus Endometrial Carcinoma (UCEC), Stomach Adenocarcinoma (STAD), Colon Adenocarcinoma (COAD), Rectum Adenocarcinoma (READ). This comprehensive overview highlights the variability in MCJ expression across different cancers, providing insights into its potential role and relevance in oncogenesis. (**A''**) The Kaplan Meier Survival curves of MCJ (DnaJC15)-low (in black) and MCJ (DnaJC15)-high (in red) in Breast cancer patients (BRCA) from The Cancer Genome Atlas (TCGA) prepared through the KM plotter. (**B-C**) Immunoblots representing protein expression levels of indicated proteins upon CRISPR-Cas9 based knock-out of MCJ ($gDNA^{MCJ}$). The sgDNA (scrambled guide DNA) served as negative control. The J-proteins Magmas and DnaJC19, which are highly related to MCJ (DnaJC15), served as internal controls. Tim23 is loading control. (**D**) Immunoblot showing shRNA mediated knock-down of MCJ. The respective cell lines being manipulated are as indicated. (**E**) Immunoblot indicating level of MCJ overexpression in different indicated cell lines against empty vector control. **(F-H)** MCF7 cells (F) or MDA-MB-231 cells (G, H) overexpressing (OE), deficient (KO) or depleted (KD) for MCJ were stained with Phalloidin and imaged using confocal microscope. Scale bar – 10 microns. **(I, J)** Relative migratory (I) and invasive (J) patterns of MDA-MB-231 cells variably expressing MCJ, Data denotes mean of n = 4 replicates. ****P(unpaired t-test)<0.0001.* **(K)** Immunoblot of MCF7 cells differentially expressing MCJ labelled with antibodies against indicated protein.

Figure S2

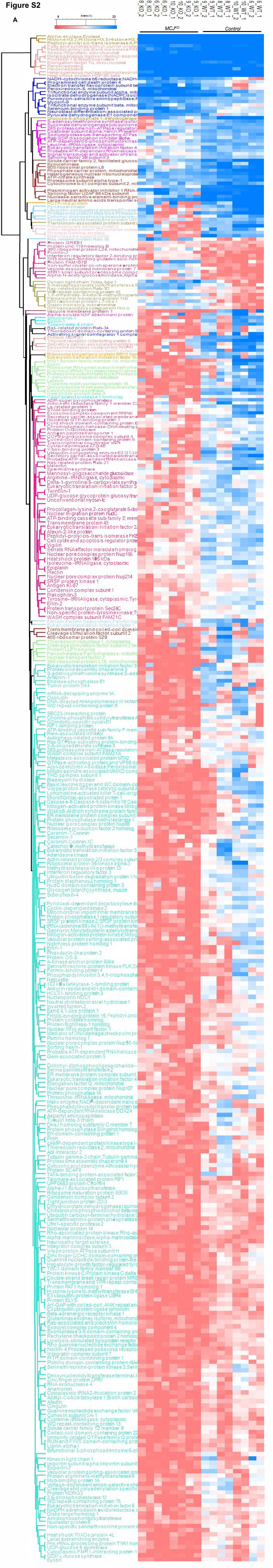

B

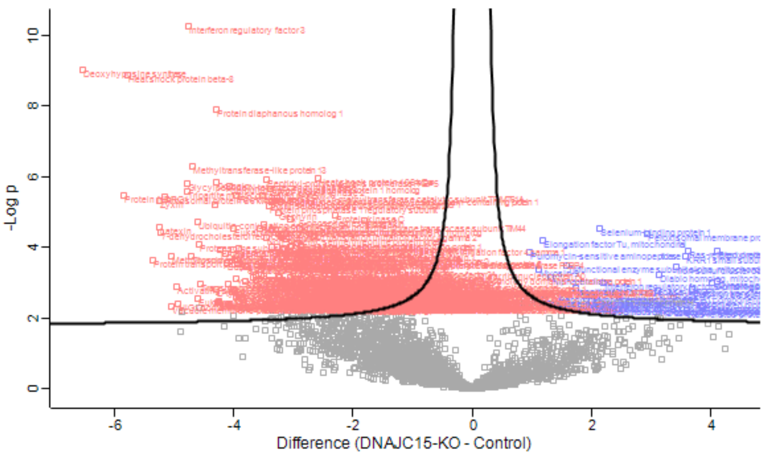

Interferon regulatory factor3
Protein diaphanous homolog 1
Methyltransferase-like protein 13
Selenium-binding protein 1
Elongation factor Tu, mitochondrial
-Log p
Difference (DNAJC15-KO - Control)

C

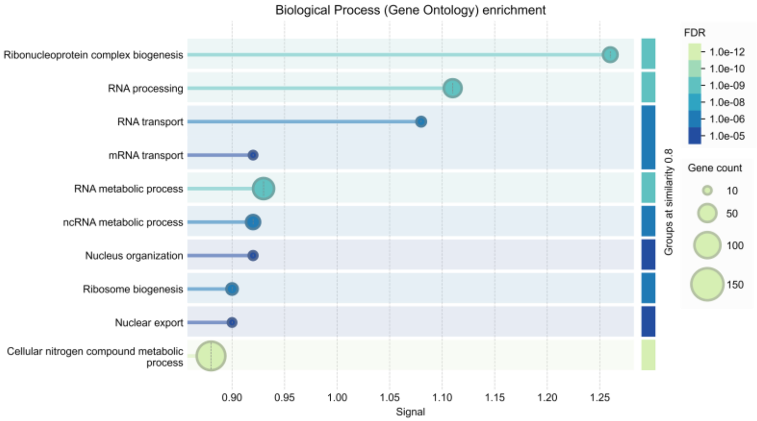

Biological Process (Gene Ontology) enrichment
Ribonucleoprotein complex biogenesis
RNA processing
RNA transport
mRNA transport
RNA metabolic process
ncRNA metabolic process
Nucleus organization
Ribosome biogenesis
Nuclear export
Cellular nitrogen compound metabolic process
Signal
Groups at similarity 0.8
FDR
1.0e-12
1.0e-10
1.0e-09
1.0e-08
1.0e-06
1.0e-05
Gene count
10
50
100
150

D

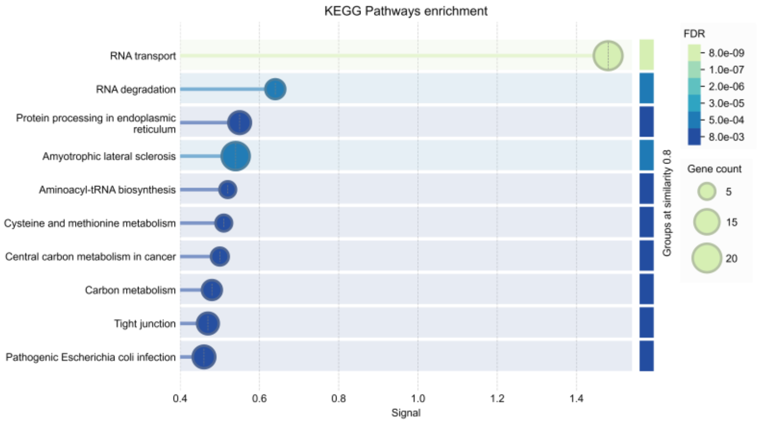

KEGG Pathways enrichment
RNA transport
RNA degradation
Protein processing in endoplasmic reticulum
Amyotrophic lateral sclerosis
Aminoacyl-tRNA biosynthesis
Cysteine and methionine metabolism
Central carbon metabolism in cancer
Carbon metabolism
Tight junction
Pathogenic Escherichia coli infection
Signal
Groups at similarity 0.8
FDR
8.0e-09
1.0e-07
2.0e-06
3.0e-05
5.0e-04
8.0e-03
Gene count
5
15
20

E

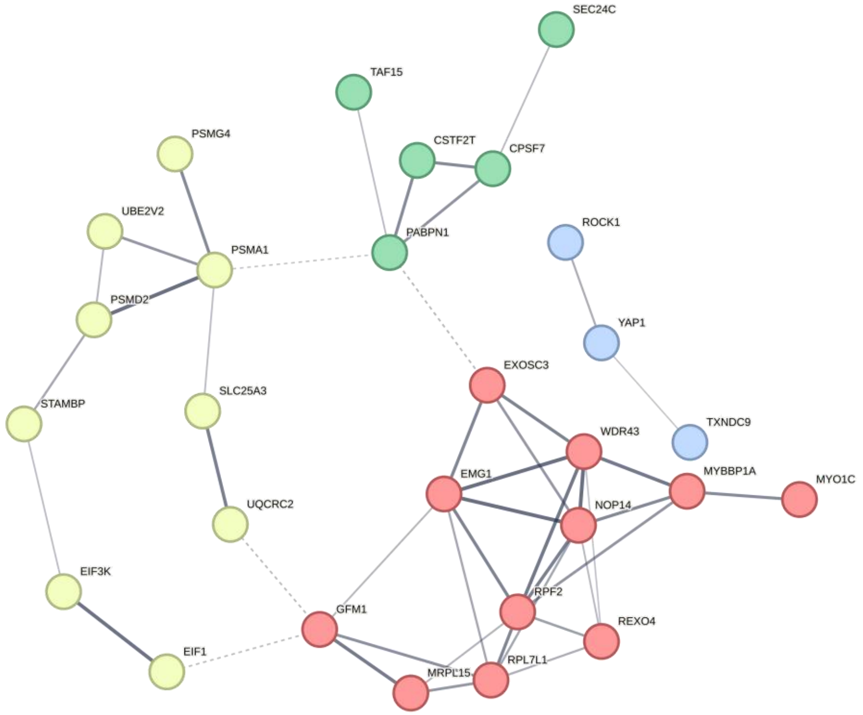

Figure. S2. (A) Heat map plot (generated with Perseus) generated with ANNOVA significant proteins (with default significance threshold with p-value<0.05) hierarchically clustered into 20 clusters representing the hierarchical clustering of proteins found to have significant differential expression between control and *MCJ*^{KO}. **(B)** Volcanic plot (generated with Perseus) of the total upregulated and downregulated protein in KO background, having an FDR of 0.05 and s0:0.1. **(C-D)** Functional enrichment of GO biological process (C) and KEGG pathways (D) grouped by similarity >=0.8 using STRING v12.0 for all the differentially expressed proteins. (**E**) PPI network of top 50 Annova-significant proteins, segregated through k-means clustering, constructed using STRING v12.0 following the default settings. PPI enrichment p-value: 0.000498.

Figure S3

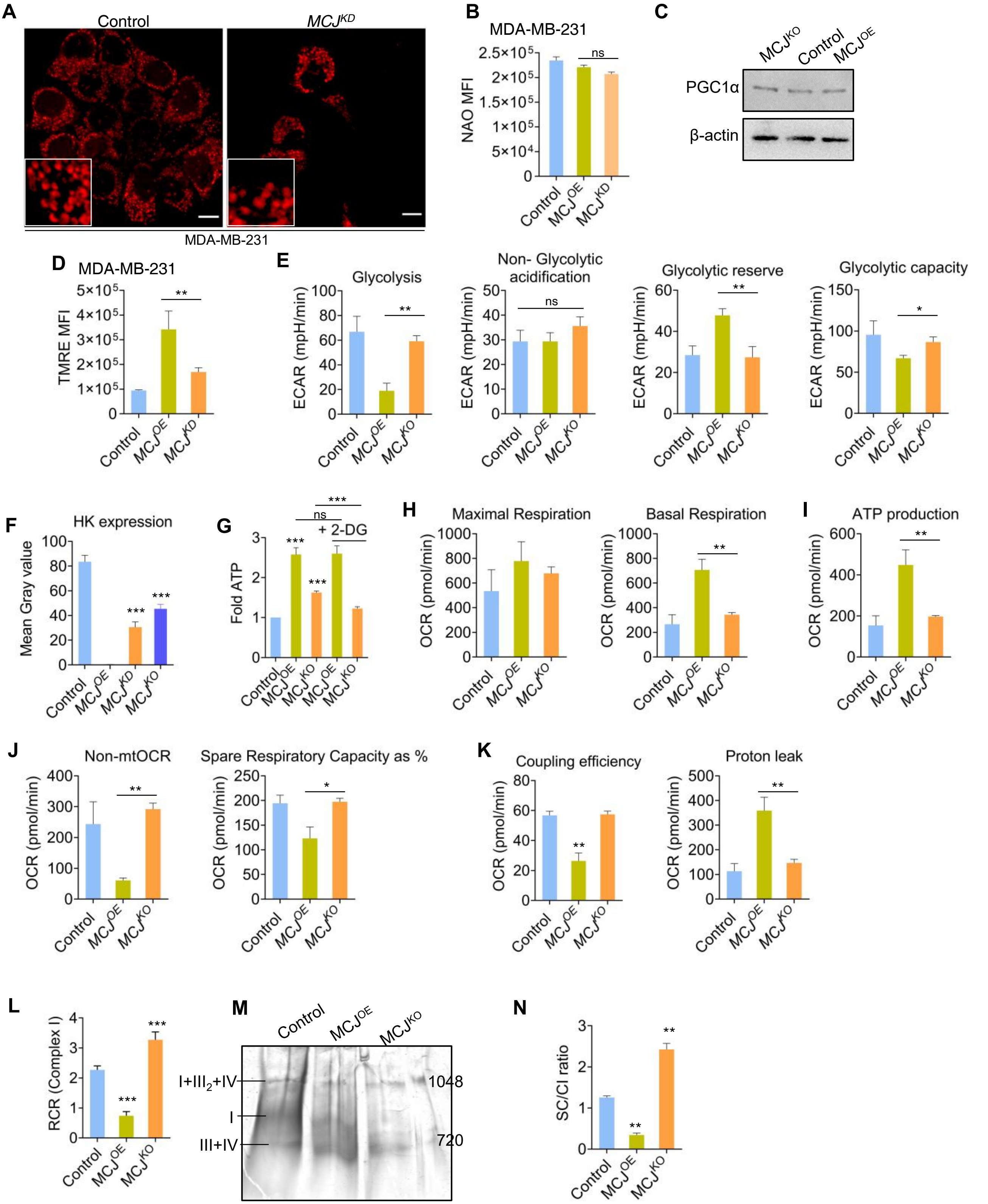

A
Control
MCJ^KD
MDA-MB-231
B
MDA-MB-231
NAO MFI
ns
Control
MCJ^OE
MCJ^KD
C
MCJ^KO
Control
MCJ^OE
PGC1α
β-actin
D
MDA-MB-231
TMRE MFI
**
E
Glycolysis
Non- Glycolytic acidification
Glycolytic reserve
Glycolytic capacity
ECAR (mpH/min)
ns
**
*
F
HK expression
Mean Gray value
***
MCJ^KO
G
Fold ATP
+ 2-DG
ns
***
H
Maximal Respiration
Basal Respiration
OCR (pmol/min)
I
ATP production
J
Non-mtOCR
Spare Respiratory Capacity as %
K
Coupling efficiency
Proton leak
L
RCR (Complex I)
M
I+III2+IV
I
III+IV
1048
720
N
SC/CI ratio

Figure S3. (**A**) MDA-MB-231 cells knocked down for MCJ were stained with MitoTracker Red and imaged using confocal microscope. Scale bar – 10 microns (**B**) N-nonyl acridine orange (NAO) staning to determine the mitochondrial mass in MDA-MB-231 cells overexpressing (OE) or depleted (KD) for MCJ, n = 4 independent replicates. Bars represent mean ± s.e.m, n = 4 replicates, ns- non-significant. (**C**) Immunoblot analysis using antibodies against PGC1α to determine levels of the protein in MCF7 cells differentially expressing MCJ. (**D**) TMRE staining to determine the mitochondrial potential in MDA-MB-231 cells overexpressing (OE) or depleted (KD) for MCJ, n = 4 independent replicates. Bars represent mean ± s.e.m, n = 4 replicates, ***P(unpaired t-test)<0.001* compared to controls. (**E**) Bar graphs representing the different glycolytic parameters as mentioned, calculated from the glycolytic stress test (Fig. 3K) denoted as mean value with s.e.m., n = 4. ***P(one-way anova)<0.001, *P(one-way anova)<0.01.* (**F**) Bar graph quantifying the levels of Hexokinase (HK) as represented in Fig. 3L. Bars represent mean ± s.e.m, n = 4 replicates, ****P(unpaired t-test)<0.0001* compared to control. (**G**) ATP levels of MCJ overexpressing (OE) and deficient cells (KO) when supplemented with 2-deoxy-glucose (2-DG), n = 4 replicates, ****P(unpaired t-test)<0.0001*. (**H-K**) Bar graphs representing the different mitochondrial respiratory parameters calculated from the mitochondrial stress test (Fig. 3M) denoted as mean value with s.e.m., n = 4. ***P(one-way anova)<0.001, *P(one-way anova)<0.01.* (**L**) Respiratory control ratio (RCR) calculated from Fig. 3M, for Complex I substrate, pyruvate. Bars represent mean ± s.e.m, n = 4 replicates, ****P(unpaired t-test)<0.0001* compared to control. All the calculations for (E), (H-K) and (L) was performed after data normalization per 10000 cells. (**M, N**) BN-PAGE of digitonin extracted mitochondrial lysate stained with Coomassie Brilliant Blue. The ratio between I-III_2-IV supercomplex (SC) vs complex I (CI) was quantified and represented as bar chart (N).

Figure S4

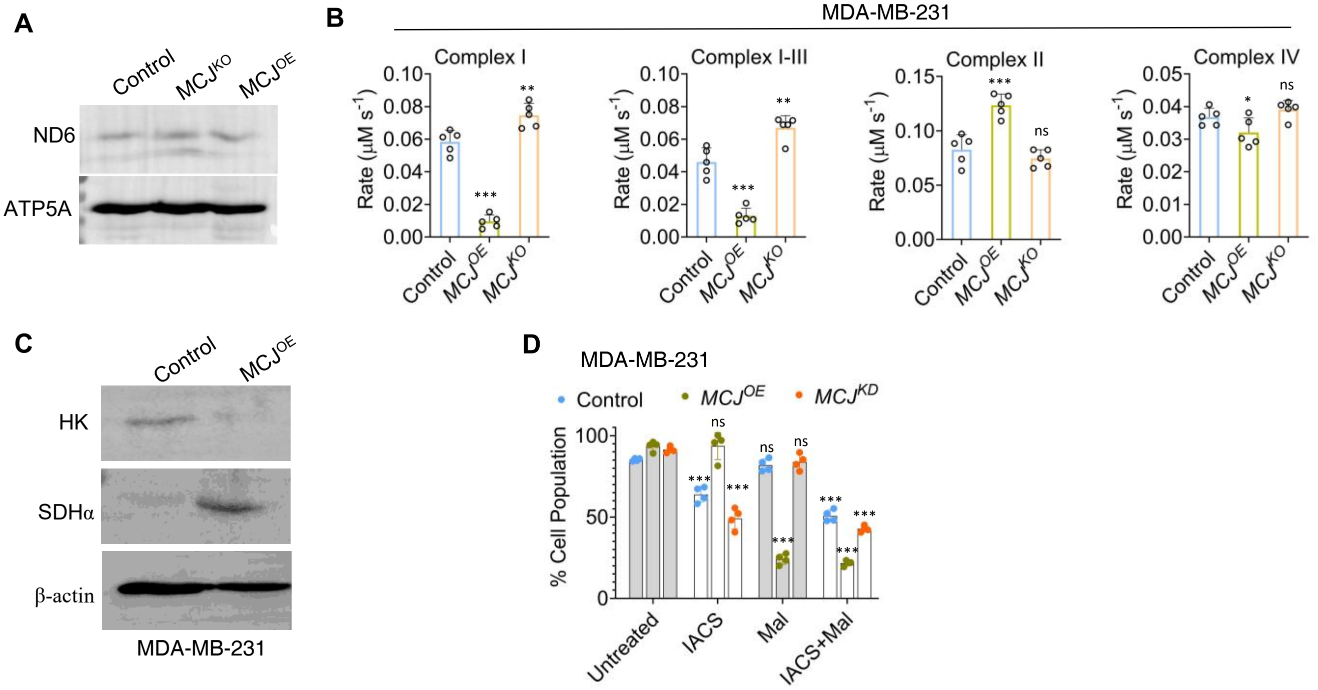

Figure S4. (**A**) Levels of Complex I subunit ND6, determined through immunoblotting, under altered levels of MCJ. ATP5A served as loading control. (**B**) Assessment of activities for Complex I, Complex II, Complex IV and transfer kinetics of Complex I-III in isolated mitochondria from MDA-MB-231 cells overexpressing MCJ or knocked-out for the protein. All enzyme activities are presented as the rate of reaction in μM/s normalized per 5 μg of protein. Bars denote mean ± s.e.m., n = 5 experiments, ****P(unpaired t-test)<0.0001,* ***P(unpaired t-test)<0.001,* **P(unpaired t-test)<0.01.* Statistical significance was calculated by comparing the activities of each genotype against control. (**C**) Immunoblot representing changes in Hexokinase (HK) and SDH levels in MDA-MB-231 cells overexpressing MCJ, compared to controls. (**D**) MDA-MB-231 cell proliferative changes induced by Complex I inhibitor, IACS-010759 (IACS), Complex II inhibitor, Malonate (Mal) either alone or in combination, determined by MTT assay. Bars denote mean ± s.e.m., n = 8, ****P(one-way annova)<0.0001*.

Figure S5

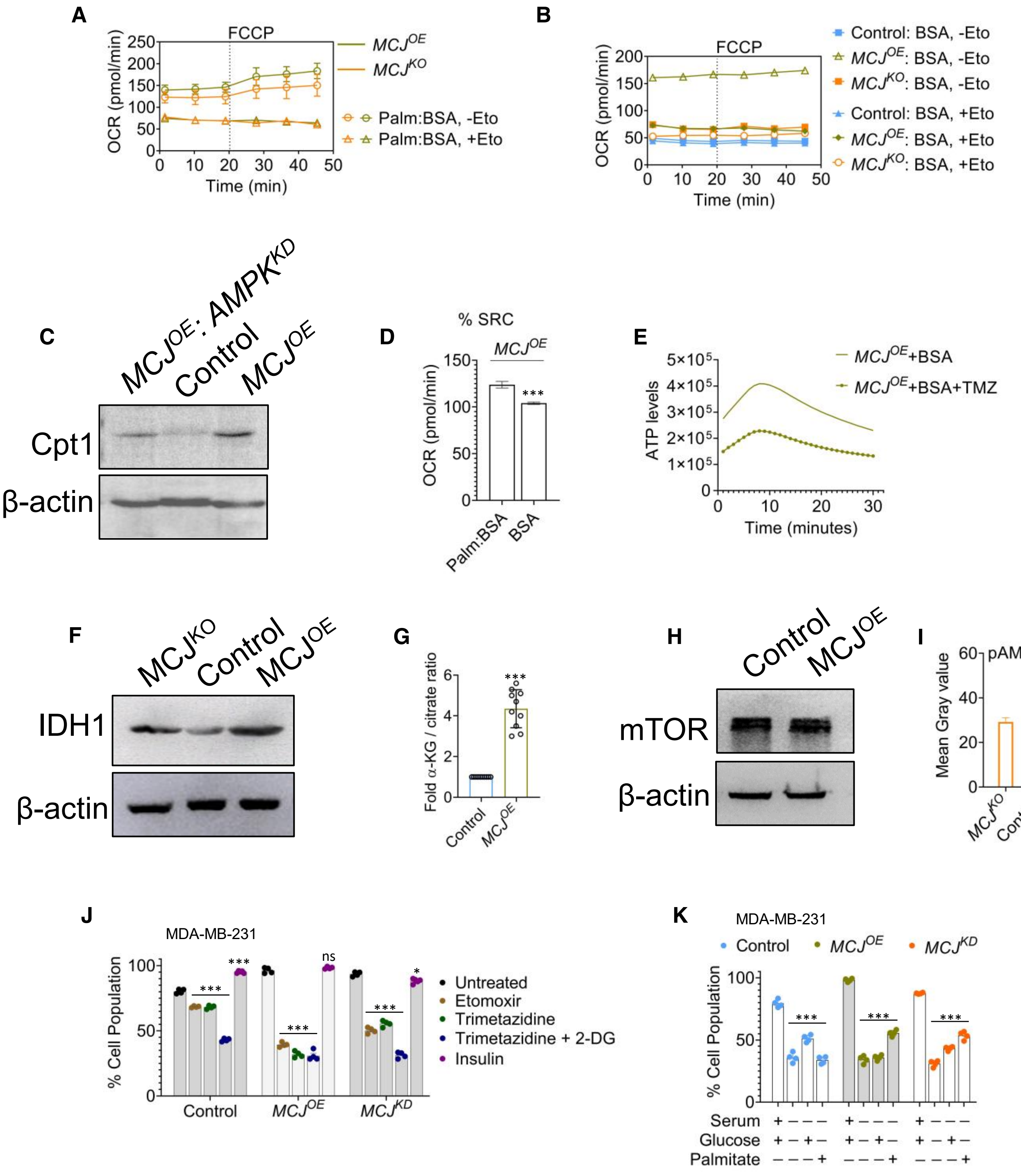

A
FCCP
OCR (pmol/min)
Time (min)
MCJ^OE
MCJ^KO
Palm:BSA, -Eto
Palm:BSA, +Eto
B
FCCP
OCR (pmol/min)
Time (min)
Control: BSA, -Eto
MCJ^OE: BSA, -Eto
MCJ^KO: BSA, -Eto
Control: BSA, +Eto
MCJ^OE: BSA, +Eto
MCJ^KO: BSA, +Eto
C
MCJ^OE: AMPK^KD
Control
MCJ^OE
Cpt1
β-actin
D
% SRC
MCJ^OE
OCR (pmol/min)
***
Palm:BSA
BSA
E
ATP levels
Time (minutes)
MCJ^OE+BSA
MCJ^OE+BSA+TMZ
F
MCJ^KO
Control
MCJ^OE
IDH1
β-actin
G
Fold α-KG / citrate ratio
***
Control
MCJ^OE
H
Control
MCJ^OE
mTOR
β-actin
I
pAMPKα
Mean Gray value
MCJ^KO
Control
MCJ^OE
J
MDA-MB-231
% Cell Population
***
ns
*
Control
MCJ^OE
MCJ^KD
Untreated
Etomoxir
Trimetazidine
Trimetazidine + 2-DG
Insulin
K
MDA-MB-231
Control
MCJ^OE
MCJ^KD
% Cell Population
***
Serum
Glucose
Palmitate

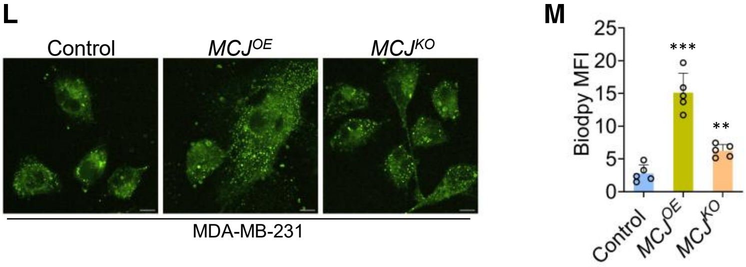

Figure. S5. (**A**) Mitochondrial OCR values for cells overexpressing (OE) or deficient (KO) for MCJ, representing the fatty acid oxidation, after addition of Palmitate conjugate (Palm)/only, BSA/Palmitate conjugate ± etomoxir to the cells following starvation for six hours. Each data points represent mean of n = 3 independent experiments, normalized per 10000 cells. *P(two-way annova)<0.0001* for OE vs KO. (**B**) Comparative mitochondrial OCR values for *MCJ*^{OE} and *MCJ*^{KO} cells in presence of BSA ± etomoxir (Eto) only. Each data points represent mean of n = 3 independent experiments, normalized per 10000 cells, *P(two-way annova)<0.0001* for OE:BSA with and without Etomoxir treatment and *P(two-way annova)<0.001* for KO. Only one FCCP injection was administered for both (A) and (B). (**C**) Levels of Cpt1 detected using specific antibody under MCJ overexpression with or without depletion of AMPK in MCF7 cells. (**D**) Spare respiratory capacity (SRC) calculated for MCJ overexpressing cells injected with Palmitate (Palm):BSA conjugate or BSA only. Bars represent mean ± s.e.m, n = 3, ****P(unpaired t-test)<0.0001*. (**E**) Mean ATP levels in cells overexpressing MCJ treated or untreated with Trimetazidine (TMZ), when supplemented with BSA only; n = 4, *P(two-way Anova)<0.0001* with respect to untreated MCJ overexpressing cells. (**F**) Immunoblot analysis of Isocitrate dehydrogenase (IDH1) in MCF7 cells with altered expression of MCJ. (**G**) Fold α-ketoglutarate (α-KG) to citrate ratio in cell overexpressing MCJ with respect to control. Bars denote mean ± s.e.m., n = 10, ****P(unpaired t-test)<0.0001*. (**H**) Immunoblot analysis of mTOR in MCF7 cells with altered expression of MCJ. (**I**) Quantification of pAMPK levels presented in Fig. 5K, depicted in form of a bar chart. (**J**) Equal number of MDA-MB-231 cells were treated with fatty acid oxidation inhibitors Etomoxir, Trimetazidine with or without glycolytic toxin 2-DG and their ability to proliferate was measured through MTT assay. Insulin that promotes lipid storage was used as an internal control. Bars denote mean ± s.e.m., n = 8, ****P(unpaired t-test)<0.0001, *P(unpaired t-test)<0.01.* (**K**) The ability of MDA-MB-231 cells to proliferate in minimal media supplemented with either glucose or palmitate, determined through MTT assay. Media enriched with serum and glucose served as control. Bars denote mean ± s.e.m., n = 8, ****P(unpaired t-test)<0.0001*. (**L-M**) Biodpy staining of lipid droplets in MDA-MB-231 cells altered for levels of MCJ expression (L), further quantified and depicted in form of a bar graph. Bars denote mean ± s.e.m., n = 5, ****P(unpaired t-test)<0.0001, **P(unpaired t-test)<0.001* compared to controls.

Figure S6

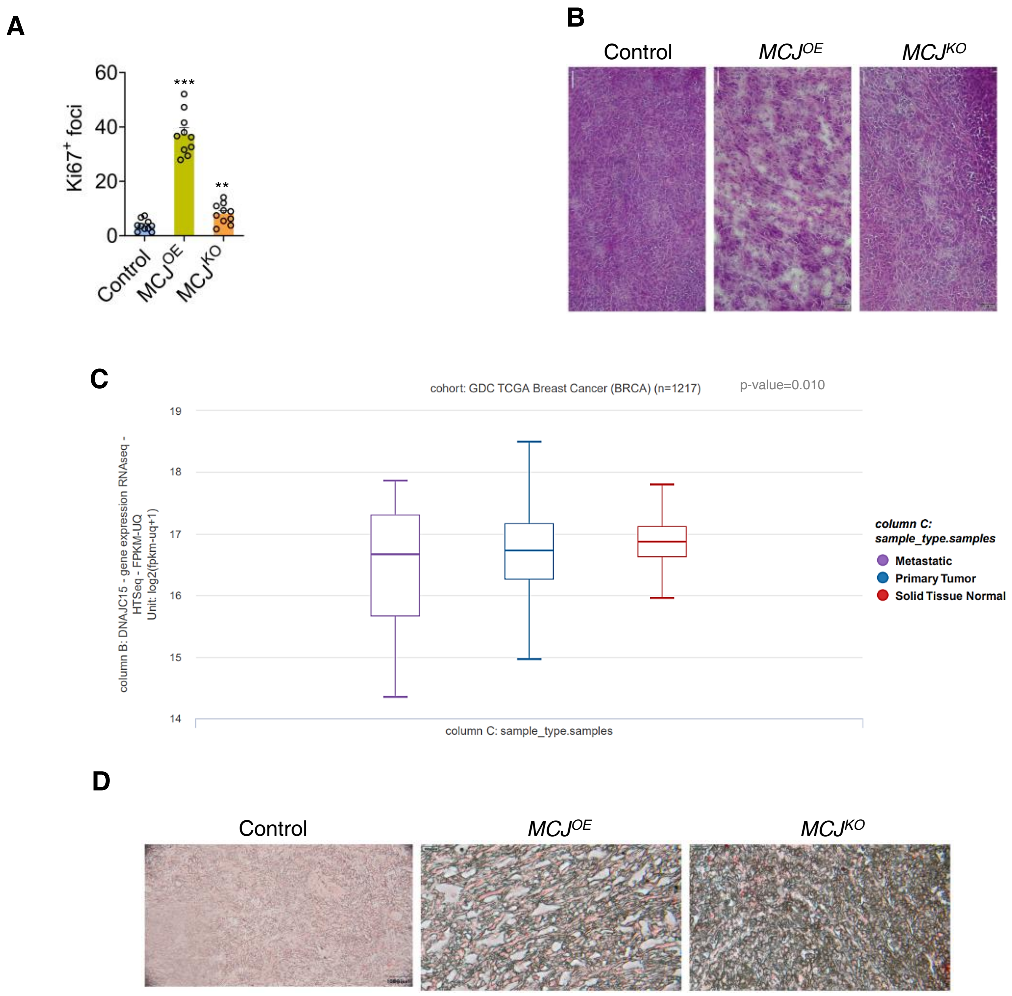

Figure S6. (**A**) The number of Ki67 foci in Fig 6C was represented as bar graph, n = 10. Each data point representing mean of 5 image slices from each group. ****P(unpaired t-test)<0.0001, **P(unpaired t-test)<0.001.* (**B**) Hematoxylin eosin staining of cryo-sectioned primary tumour from each group depicting the collective tissue migration. (**C**) The TGCA data base shows the MCJ (DnaJC15) expression (RNA-seq) in breast cancer tissues ("Primary Tumor", n = 1097), ("Metastatic", n = 7) and normal tissues ("Solid tissue normal", n = 113); *P*-value(dataset)=0.01066 (f= 4.558) as calculated with One-way ANNOVA. (**D**) Primary tumour sections stained with Oil Red O to show the accumulation of cytoplasmic lipid droplets (pink colour). Scale bar – 100 pixels.

Figure S7: Uncropped Western Blots

Figure 3L

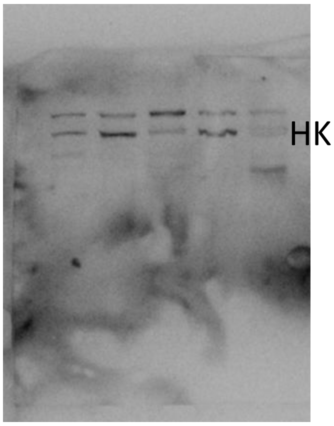

Figure 4B

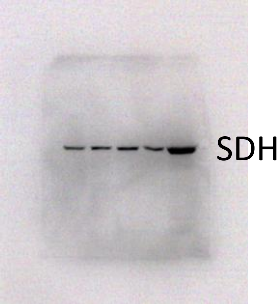

Figure 5D

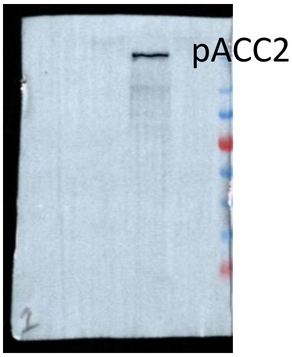

Figure 5K

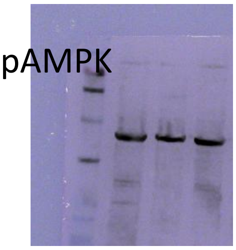

Figure 6F

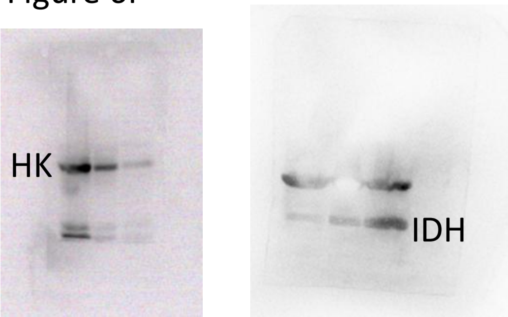

qPCR data

Figure 5C

		MCJ^{KO}			Cont			MCJ^{OE}		
cpt1		1.46	0.87	2.41	1	1	1	1.755	1.98	1.641
ACC2		0.856	0.741	0.953	1	1	1	4.983	4.125	5.617
ACLY		0.385	0.296	0.405	1	1	1	1.42	1.382	1.58
FASN		0.55	0.41	0.67	1	1	1	1.94	1.89	2.09

Figure 5L

		MCJ^{KO}			Cont			MCJ^{OE}			
Cdk1		0.27	0.152	0.346	1	1	1	6.4	5.12	7.17	
C-MYC		4.616	2.145	5.974	1	1	1	7.64	8.17	6.32	
