# Supplementary material for: MCJ modulates mitochondrial ETC flux to promote lipid metabolism–driven enhancement of cell proliferation and migration
Source: Cell Death Dis. 2026 Jan 2;17(1):151. doi: 10.1038/s41419-025-08398-y (PMC12859062; doi:10.1038/s41419-025-08398-y)
Supplement: Supplementary file 2 — Supplemental Methods [file 41419_2025_8398_MOESM2_ESM.pdf]

Supplemental Methods:

Material and Methods

Cell lines and culture

MCF-7 and MDA-MB- 231 breast cancer cell lines were obtained from National Centre for Cell Science, Pune, India. The 4T1-Luc2 cells were kind gift from Dr. Dipak Dutta, CDRI, Lucknow. All cell lines were grown in a monolayer. MCF7 was maintained in Eagle's Minimum Essential Medium (EMEM), MDA-MB- 231 in Leibovitz's L-15 media and 4T1-Luc2 in RPMI-1640 (Gibco), all supplemented with 10% fetal bovine serum (FBS), 100 U/ml penicillin, 100 µg/ml streptomycin (Gibco). The cells were grown in 5% CO_2 in a humidified incubator at 37^0 C.

MCJ overexpression lines were generated by transfecting the MCF7, MDA-MB- 231 or 4T1-Luc2 cells with pCIneo plasmid (Promega) containing *MCJ* gene insert (OriGene). The positive colonies were expanded with G418 selection (800 µg/ml) after transfection. 20 such clonal population were mixed, protein overexpression verified through western blot and the mixed population MCJ^{OE} cells were maintained in 200 µg/ml G418 for further downstream experiments.

MCJ knockout cell lines (MCJ^{KO}) were generated in MCF7 and 4T1-Luc2 by electroporating the cells with Cas9 protein and guide RNA (gRNA) targeting exon 1 of the *MCJ (DNAJC15)* gene, in specified ratio according to the manufacturer's protocol. Post- transfection, 20 single cell colonies were amplified after transfection, mixed together and verified for loss of MCJ protein expression by western blot. The gRNA selected did not possess any off targets. To further nullify the possibility of off-target effect, expression levels of two related highly homologous genes, *DNAJC19* and *MAGMAS* was tested.

MCJ knockdown lines (MCJ^{KD}) in MDA-MB-231 were generated in pLKO.1 - TRC cloning vector according to the corresponding protocol in Addgene. The 21mers used were as follows: Forward - 5' CCGG GCTAAGATTAGAACAGCTCAT CTCGAG ATGAGCTGTTCTAATCTTAGC TTTTTG 3' and Reverse - 5' AATTCAAAAA GCTAAGATTAGAACAGCTCAT CTCGAG ATGAGCTGTTCTAATCTTAGC 3'. Cellular transfection was performed using electroporation and 20 positive clones were selected in 2 µg/ml Puromycin, mixed together, followed by testing the depletion of protein expression with western blotting. The stable line was further maintained in 0.5 µg/ml puromycin.

Estimation of NAD^+ and NADH

The estimation of cellular NAD+ and NADH was performed using NAD/NADH-Glo™ Assay according to the manufacturer's protocol (Promega: NAD/NADH-Glo™ Assay Instructions for Products G9071 and G9072). Briefly, the cells were plated in a white-walled tissue culture plate. After 24 hours the plate was equilibrated at room temperature for 5 minutes. 100 µl of NAD/NADH-Glo™ Detection Reagent with or without reductase enzyme was added to each well and incubated for 30 minutes at room temperature. The luminescence was recorded using a Spectramax iD5 (Molecular Devices) and the graph was plotted using GraphPad Prism 8.

In case of tumour grafts, snap frozen primary tumours, weighed in equal amount, were homogenized in cell lysis buffer. 100 µl of the homogenate wt/vol were taken in 96-well plate and equivalent volume of NAD/NADH-Glo™ Detection Reagent without reductase enzyme was added. The plate was incubated for 30 minutes at room temperature. The luminescence was recorded using an IVIS Spectrum (Perkin Elmer) and the graph was plotted using GraphPad Prism 8.

Determination of cellular ATP content

For measurement of ATP levels, Mitochondrial ToxGlo ATP Assay (Promega: G8000) was performed according to the manufacturer's protocol. The cells seeded in 96 well plates were incubated at 37°C in a humidified and CO2-supplemented incubator. After 24 hours, 20 µl of the 5X Cytotoxicity Reagent was added to each well. The mixture was briefly mixed for 1 minute by orbital shaking at a speed of 500-700 rpm to ensure proper mixing of the reagent and sample and minimise potential partitioning. The plate was then incubated at 37°C for 30 minutes. After the incubation, fluorescence measurements were taken at an excitation wavelength of 485 nm and an emission wavelength range of 520-530 nm. The signal was adjusted to be 60-90% of detector saturation to achieve optimal results. Finally, the assay plate was allowed to equilibrate to room temperature for 5-10 minutes. Then, 100 µl of ATP Detection Reagent was added to each well, and the plate was mixed by orbital shaking at a speed of 500-700 rpm for 1-5 minutes. The luminescence was measured with Spectramax iD5 (Molecular Devices).

In case of Etomoxir or Trimetazidine treatment, the seeded cells were treated with either 2 μM Etomoxir or 5 μM Trimetazidine for 6 h, followed by detection of ATP levels as per protocol mentioned above.

In case of tumour grafts, snap frozen primary tumour weighed equally, were homogenized in cell lysis buffer. 100 μl of the homogenate wt/vol was taken and equivalent volume of ATP Detection Reagent was added. The plate was mixed by orbital shaking at a speed of 500-700 rpm for 1-5 minutes and the luminescence was measured with an IVIS Spectrum (Perkin Elmer).

Glycolytic Stress Test

The test was performed according to the Agilent Seahorse XF Glycolysis Stress Test Kit in every detail. Cells were plated at 40,000 cells per well in Seahorse XF cell culture microplates. The sensor cartridge was kept at 37^0C non-CO_2 incubator overnight for hydration. After 24 hours the culture plate was checked for optimal confluency and the media was changed to warm assay media. The plate was then kept at 37^0C non-CO_2 incubator for one hour. Acquisition was then done according to the instructions of the manufacturer. During the assay Glucose, Oligomycin and 2-DG were sequentially injected at final concentrations of 10 mM, 1 μM and 50 mM respectively.

Mitochondrial Stress Test and assaying for Electron Flow

The test was performed according to the Agilent Seahorse XF Mito Stress Test Kit in each detail. Optimal FCCP concentration was determined by titration and was found to be 0.5 μM. The sensor cartridge was kept at 37^0C non-CO_2 incubator overnight for hydration. 40,000 cells per well were seeded in Seahorse XF cell culture microplates and kept at 37^0C CO_2 incubator overnight. The culture plate was checked after 24 hours for optimal confluency and the media was changed to warm assay media with addition of 1 mM pyruvate, 2 mM glutamine and 10 mM glucose. Acquisition was made according to the instructions of the manufacturer. During the assay the following reagents were injected to their final concentrations in a sequence mentioned in the respective figures – 1.5 μM Oligomycin, 0.5 μM FCCP, 0.5 μM Rotenone, 0.5μM Antimycin. The electron flow assay was performed using 40,000 cells per well with initial presence of 0.5 μM FCCP. The injection port was filled to their final concentrations in

accordance to the experiment with final either with 2 μM Rotenone, 2 mM Malonate, 4 μM Antimycin A, 10 mM Ascorbate+ 100 μM TMPD (N1, N1, N1, N1-tetramethyl-1,4-phenylene diamine) or 20 mM sodium azide. All the reagents were freshly prepared prior to the assay.

Quantification of Respiratory Chain Complexes activity

Complex I activity was assessed by incubating isolated mitochondria in phosphate buffer supplemented with 1% deoxycholate (DOC) to achieve membrane permeabilization and 2 mM sodium azide for 15 mins on ice. Subsequently, permeabilized mitochondria were incubated with 1 mM NADH, and the reaction was initiated by adding 60 μM ubiquinone. The oxidation of NADH was monitored as a decline in absorbance at 340 nm for 2 min. For Complex I-III activity sodium azide treated permeabilized mitochondria were incubated with 1 mM NADH and the reaction was initiated by adding 65 μM cytochrome c. The reduction of cytochrome c was detected as an increase in absorbance at 550 nm. Complex II activity was evaluated using a coupled enzymatic assay. Mitochondria were first incubated with 20 mM succinate in phosphate buffer at 30°C for 10 min to activate the enzyme. To selectively measure complex II, 2 μg/μl rotenone, 2 μg/μl antimycin, and 2 mM sodium azide were added to inhibit complexes I, III, and IV, respectively. The reaction was initiated by adding 65 μM ubiquinone, and the resulting reduction of ubiquinone was coupled to the reduction of 50 μM DCPIP. The conversion of DCPIP to its colorless reduced form was tracked by recording the decrease in absorbance at 600 nm. Complex IV activity was assessed by measuring the decrease in absorbance at 550 nm corresponding to the oxidation of reduced cytochrome *c*. A parallel reaction was set up for every experiment with the inhibitor of respective complexes to assure the specificity of the reaction.

Fatty acid oxidation induced OCR

The test was performed according to the Agilent Seahorse XF Mito Stress Test Kit with modification according the instructions in the manual. 40,000 cells were seeded per well in Seahorse XF cell culture microplates. The sensor cartridge was kept at 37^0C non-CO2 incubator overnight for hydration. After 24 h, the culture plate was checked for optimal confluency and the media was changed to substrate limiting media consisting of Seahorse assay media with 1 mM glutamine, 0.5 mM L-carnitine and 1% FBS where the cells were allowed to

equilibrate for next six hours. Only FCCP injection (Final conc. 0.5 μM) was included to evaluate the baseline and maximal fatty acid oxidation mediated OCR. Etomoxir was added 15 mins prior, followed by addition of substrates in defined order as indicated in the figures and then proceeded with the acquisition. The final concentrations of the substrates used were - 150 μM Palmitate-BSA conjugate, 150 μM BSA and 2 μM Etomoxir.

Amino acid induced OCR:

40,000 cells were seeded per well in Seahorse XF cell culture microplates and parallelly, the sensor cartridge was kept at 37^{0}C non-CO2 incubator overnight for hydration. The culture plate was checked for optimal confluency and the media was changed to substrate limiting media consisting of seahorse assay media with 1mM glutamine and 0.5mM L-carnitine in which the cells were incubated for the next six hours. Only FCCP injection (Final conc. 0.5 μM) was included to evaluate the baseline and maximal OCR. The amino acid mixture (1x Gibco MEM NEAA) as indicated in the figure was added prior to acquisition. Citrate and α-ketoglutarate levels were estimated using Citrate Assay Kit (MAK333) and α-Ketoglutarate Quantitation Kit (MAK541) (Sigma-Aldrich) respectively.

Lipid staining

Seeded cultured cells were rinsed with 1X PBS before staining with 0.5% Oil Red O or 2 μM BODIPY™ 493/503 for 15 min, followed by multiple washes in 1X PBS. Image acquisition for Oil Red O was performed under bright field microscope (Olympus BX53) and for BODIPY™, live-cell images were acquired through confocal microscopy.

Paraffin embedded primary tumour sections were kept in incubator at 65^{0}C for one hour and then incubated in xylene three times for 10 mins each. The sections were air dried till it became whitish and dipped in 100% alcohol for 5 mins. The samples were rehydrated in alcohol gradient 100%-90%-70% for 5 mins each. After 70% alcohol treatment, the sections were stained in 0.5% Oil O Red stain prepared in 70% isopropanol for 20 mins, followed by multiple washes before image acquisition under bright field microscope (Olympus BX53).

Cell Migration and Invasion

The analysis of cell migration was performed using the Boyden chamber experiment. Cell culture inserts with membrane pore size of 8 µm constituted the upper compartment. The cells were placed in the upper compartment in serum deficient media and serum was used as a chemoattractant in the lower chamber. The cells were then incubated for 24 hours. After the 24-hour incubation period, the inserts were removed from the plates. The upper side of the membrane was gently cleaned using a cotton swab, to remove any remaining cells on that side. The rate at which cells migrated from one side of the membrane to the other in search of better growth conditions determined their migration capability. The cells that had migrated to the lower side of the membrane were fixed using methanol, stained with crystal violet, quantified and plotted using GraphPad Prism 8. For studying cell invasion, experiment was conducted in a manner identical to the cell migration assay, except that the insert's membrane in this assay was coated with Geltrex (ThermoFisher Scientific), mimicking the presence of a basement membrane.

The migratory ability of the cells upon exposure to different respiratory complex inhibitors was performed as mentioned above except that the cells placed in upper compartment were exposed to either 2 mM Malonate, 1.4 nM IACS-010759 or both. For carbon source supplementation experiments, the cells were placed in minimal media either devoid of glucose or supplemented with 8 mM glucose or 50 µM palmitate.

Estimation of the Superoxide levels

After 24 hours of seeding, the cells were incubated with 0.05 µM MitoSOX Red (ThermoFisher Scientific) for 15 minutes. Cells were then washed twice with 1X PBS prior to acquisition of fluorescence at 610 nm in Synergy H1 microplate reader (version 2.09.1). The fluorescence intensity was quantified and plotted using GraphPad Prism 8.

Mitochondrial membrane potential

0.05 µM Tetramethylrhodamine ethyl ester (TMRE) (ThermoFisher Scientific) was added to the cells and incubated for 15 minutes. Following incubation, the cells were washed three times with PBS to remove any unbound dye or debris. Subsequently, FluroBrite DMEM (Dulbecco's Modified Eagle Medium) was added to the wells. The fluorescence emitted by the TMRE dye-labelled mitochondria was then measured at 574 nm using Synergy H1 microplate reader

(version 2.09.1). The fluorescence intensity was quantified and plotted using GraphPad Prism 8.

Determination of Mitochondrial mass and morphology

Mitochondrial mass was determined by staining the cells with NAO (ThermoFisher Scientific) for fifteen minutes. After the incubation period, the NAO solution was removed and the cells were washed with 1x PBS prior to detection of fluorescence using the Synergy H1 microplate reader (version 2.09.1). The fluorescence intensity was quantified and plotted using GraphPad Prism 8. For mitochondrial morphology, the cells were incubated with MitoTracker CMXROS (ThermoFisher Scientific) for 15 mins. Cells were washed with 1x PBS and mounted in Prolong gold antifade reagent (ThermoFisher Scientific). Images were acquired using Zeiss LSM 900 microscope and processed using Adobe Photoshop 2021.

Transmission electron microscopy

The cells were fixed in a solution containing 0.1 M HEPES/KOH (pH 7.2), 4 mM CaCl2, and 2.5% glutaraldehyde for 4 hours at room temperature. Subsequently, the cells underwent three rinses with 0.1 M HEPES/KOH (pH 7.2), 4 mM CaCl2, and were post-fixed in 1% osmium tetroxide for 45 minutes at 4°C. After 48 hours and three rinses in distilled water, the cells were treated with 1% uranyl acetate for 1 hour at 4°C. The samples were dehydrated through a graduated ethanol series, infiltrated with Epon, and embedded using standard procedures. Ultrathin sections (40–70 nm) were cut and mounted on copper grids. Staining was performed, and the sections were examined with a Zeiss CEM 902 transmission electron microscope (Carl Zeiss) at 80 kV. The visible cristae and mitochondrial perimeter were manually demarcated, and the respective area was measured using ImageJ (FIJI).

Immunostaining

Primary tumour sections embedded in paraffin were deparaffinized in an incubator at 65^0C for one hour after which it was dipped in xylene three times for 10 mins each. The sections were air dried, dipped in 100% alcohol for 5 mins, and kept to rehydrate in alcohol gradient 100%-90%-70%-50%-30% for 5 mins each. Post 1x TBS and sodium citrate wash, the samples were heated at 100^0C for 15 mins and left to cool in sodium citrate buffer to room temperature, followed by further 1X TBS and H_2O_2 wash for 20 mins. H_2O_2 was washed off and slides were

incubated with blocking (1% BSA) for 1 h at room temperature, followed by primary antibody incubation, 0.5% TBST wash and labelling with Alexa Flour 633 conjugated secondary antibody for 2 hours at room temperature. The samples were washed with 0.5% TBST prior to mounting with DABCO. The imaging was performed using ZEISS LSM900, processed with ImageJ (FIJI) and panels were prepared in Adobe Photoshop 2021.

Sample preparation and Proteomics analysis

The cell lysate prepared in Urea lysis buffer (7M Urea, 2M thiourea, 4% CHAPS, 10mM Tris, 65mM dithiothreitol (DTT) & 0.1mM PMSF) was sonicated on ice and centrifuged at 13,000x g for 15 minutes at 4°C. The supernatant was collected was precipitated using the TCA-acetone precipitation method, with the lysate, and absolute TCA in a ratio of 1:3. The mixture was then incubated at -20°C for 1 hour and centrifuged at 13,000xg for 20 minutes at 4°C. The resulting pellets was chilled absolute acetone, air-dried, and stored at -80°C until needed.

For in-solution protein digestion, the protein pellet was re-solubilized in a urea solution (1X RapiGest and 100 mM ammonium bicarbonate [ABC]), and its concentration was determined using a BCA kit. Subsequently, 50 µg of purified protein samples were taken, reduced with DTT, and alkylated with iodoacetamide (IAA). Alkylation was quenched with DTT. MS-grade trypsin (Cat #90058, Pierce) was added to the reaction mixture at an enzyme-to-protein ratio of 1:40, allowing for protein trypsinization for 16 hours at 37°C. The reaction was stopped by drying the mixture in a vacuum concentrator. The resulting dried peptides were reconstituted in 0.1% formic acid for desalting using C-18 columns (Cat #89870, Pierce). Desalting was performed with 3 washed with 1% formic acid and the peptides were eluted with 1% FA + ACN (acetonitrile). Peptides were vacuum dried and stored at -80°C until further use.

For label-free LC-MS/MS analysis, desalted lyophilized peptide samples were reconstituted with 0.1% (v/v) formic acid (FA) in MilliQ water. The peptide concentration was determined using Nanodrop with its Protein A205/280. Subsequently, 1 µg of peptides was loaded onto the LC column and separated using an LC gradient consisting of 80% ACN and 0.1% FA over 120 minutes. MS analysis was carried out using a TIMS/TOF Pro Mass Spectrometer (Burker). Bovine serum albumin (BSA) was included at the beginning and end of each MS run to assess instrument quality. Mass spectrometric data acquisition was performed in data-dependent acquisition mode. The protein search was executed against the *Homo sapiens* database, obtained from UniProt. Trypsin was designated as the protease. Raw data was processed in MaxQuant software. A false discovery rate of 1% was applied at both the peptide-spectrum

match (PSM) and of 5% at protein levels. Data integrity check, annotation, normalization, and statistical analysis of identified proteins were carried out using Perseus software. Enrichment analysis of biological pathways and processes for significantly dysregulated proteins was conducted using Metascape (version 3.5.20230101). The analysis incorporated ontology sources such as GO Biological Processes, KEGG Pathway, Reactome Gene Sets, CORUM, WikiPathways, and PANTHER Pathway. Protein–protein interaction (PPI) analysis was performed on ANOVA-significant proteins using STRING v12 with default settings. The resulting network was subsequently analyzed to identify functionally enriched GO biological processes as well as Reactome/KEGG pathway enrichment.

RNA isolation

RNA was extracted from genetically modified cell lines using the Qiagen Miniprep Isolation Kit. The cell lysate was homogenized by pipetting, and then an equal volume of 70% ethanol was added. The mixture was loaded onto RNeasy spin column, followed by DNase treatment in the column for 15 minutes. After several alcohol washes, the DNase-treated RNA was collected and subsequently quantified using a Nanodrop spectrophotometer (ThermoFisher Scientific).

Quantitative Real time PCR

A semi-quantitative transcript analysis was conducted by synthesis of first-strand cDNA with 5 µg of total RNA, along with 200 U reverse transcriptase (ThermoFisher Scientific), 20 U RNase inhibitor (Ribolock, Thermo Fisher), 10 mM dNTP mix, and random hexamer. The RNA and 2 µl of random hexamer were incubated at 65°C for 5 minutes, followed by chilling on ice. The remaining components, pre-mixed in the order of 5x reaction buffer, Ribolock RNase inhibitor, 10 mM dNTPs, and reverse transcriptase, were then added. The cDNA synthesis involved a PCR program with temperature steps at 25°C for 5 minutes, 42°C for 60 minutes, 70°C for 10 minutes, and a final hold at 4°C. Finally, 0.5 µl of the produced cDNA was employed for quantitative PCR, utilising the following components: SYBR Green qPCR mix (Puregene), gene-specific primers, and water. Graphs were plotted as fold change in expression determined using the $2^{-\Delta.\Delta Ct}$ method.

Immunoblotting

The cells were lysed by adding 1x RIPA buffer. The resulting lysate or the homogenized tissue samples were centrifuged at 12,000 rpm for 20 minutes to pellet the cell debris. The protein content in the supernatant was determined using the Bradford reagent (BioRad).

The cell lysate was combined with Lamelli buffer, which consisted of 250 mM Tris-Cl (pH 6.8), 500 mM dithiothreitol, 10% SDS, 1% bromophenol blue, and 50% glycerol. The mixture was then heated at 100°C for 10 minutes. The samples were then centrifuged at 12,000 rpm at 4°C for 10 minutes prior to resolving in SDS-Polyacrylamide gel. The resolved proteins were electro-transferred to a PVDF membrane (Merk Millipore). The membrane was blocked with γ-globulin free BSA (Sigma-Merck) and immunoblotted using indicated primary antibodies. Secondary detection was performed using horseradish peroxidase (HRP) conjugated anti-rabbit or anti-mouse secondary antibodies (GE Amersham (Cytiva)) at dilution of 1:20000 or 1:10000 respectively. Finally, the protein bands on the membrane were detected using an enhanced chemiluminescence (ECL) substrate in the Vilber Lourmat Chemidoc system.

Blue-Native PAGE

The assay was essentially carried out as mentioned *(21)*. Briefly, 400 μg of isolated mitochondria from each genotype was solubilized in 1% digitonin containing buffer by incubating for 10 mins and centrifuged at 20,000 x g for 10 min. The samples were suspended in 0.2% Coomassie blue G-250 and 5 mM aminocaproic acid, followed by resolving the complexes blue-native gradient gel. After electrophoresis, the gel was stained with 0.2% Coomassie blue R-250 and the bands were quantified using ImageJ.

Determination of Cell Proliferation

EdU (5-ethynyl-2'-deoxyuridine) (Abcam), a thymidine analogue was used in this assay for measuring the DNA proliferation rate. The procedure was done according to the manufacturer's protocol. Cells were treated with EdU 20 h post-seeding. After 24 h, the cells were trypsinized and washed with 1X PBS, followed by fixation in 4% PFA and permeabilization using Permeabilization Buffer. EdU Additive Solution containing iFluor-488 was then added to the cells and incubated for 30 min to allow it to covalently cross-link with the EdU incorporated

in the DNA. The cells were then washed with 1XPBS and data was acquired using flow cytometry.

The relative proliferation of the cells under treatment or supplemented growth media conditions was performed using MTT assay. After 24 h post-seeding, the cells were treated with either 2 µM Etomoxir, 5 µM Trimetazidine, 0.12 IU/µL Insulin, 2 mM Malonate or 1.4 nM IACS-010759. The relative cell population was evaluated through MTT assay, 24 h post-treatment.

To study the effect of different substrates on cell proliferation and migration, the cells were serum starved in minimal media for 24 h, followed by evaluation of their growth in minimal media supplemented by either 8 mM glucose or 150 µM palmitate.

Phalloidin and DAPI Staining

Cells initially seeded onto glass coverslips, were subjected to three washes of chilled 1x PBS. Following the washes, the cells were fixed with a 4% formaldehyde solution in 1x PBS for a duration of 10 minutes. To permeabilize the cells, 0.5% Triton X-100 solution in 1x PBS was applied at room temperature for 10 minutes. Once permeabilization was complete, the cells were washed with 0.1% PBST (PBS containing 0.1% Tween 20) solution. Next, the cells were incubated overnight at 4 ^{o}C with Phalloidin, diluted at a ratio of 1:1000. Cells were then washed three times for 5 min each using 0.1% PBST. Subsequently, the cells were incubated with DAPI for 10 minutes at room temperature. After the incubation, the cells were washed again with 1x PBS and mounted using DABCO (a mounting medium). The imaging was done using Zeiss LSM900, processed with ImageJ (FIJI) and panels were prepared in Adobe Photoshop 2021.

Metadata analysis

MCJ (DNAJC15) transcript analysis in breast cancer based on the sample type was achieved with cancer genome atlas computational tool; Xena. GDC TGCA breast cancer (BRCA) study was conducted with selections; DNAJC15 as genomic variable and sample type as phenotypic variable for gene expression studies with this data set.

Expression correlation studies with patient survival was performed with Kaplan-Meier Plotter utilizing TGCA RNA-seq database, accessible at https://portal.gdc.cancer.gov/.

Pan Cancer expression analysis were performed with similar protocol utilizing UCSC Xena browser with the GDC TCGA Pan-Cancer (PANCAN) dataset. Expression levels were obtained for different cancer types, including primary tumors, metastatic tumors, recurrent

tumors and solid tissue normal samples. The analysis included diverse cancers such as breast invasive carcinoma (BRCA), lung adenocarcinoma (LUAD), prostate adenocarcinoma (PRAD), and colon adenocarcinoma (COAD) etc. Box plots and bar charts visualized DNAJC15 expression levels, and statistical analysis determined the significance of differences between cancer types and between cancerous and normal tissues.

Correlation analysis was performed to evaluate the relationship between MCJ (DNAJC15) and SDHA expression levels across different datasets. Expression data were obtained from TCGA tumor, TCGA normal, and GTEx breast tissue datasets using the Gene Expression Profiling Interactive Analysis (GEPIA) tool. The data were log2-transformed and expressed as transcripts per million (TPM). The Pearson correlation coefficient (R) and p-value were calculated using GEPIA to determine the strength and significance of the correlation. A scatter plot was generated to visualize the correlation.

Animal studies

All the experiments were approved by the Institutional animal ethics committee of ACTREC, TMC, India, and were performed in accordance with accepted guidelines. For in vivo non-invasive imaging, 0.5×10^6 firefly luciferase reporter labelled 4T1Luc2 cells overexpressing or deficient for MCJ along with control, were implanted in mammary fat-pad of 6–8 weeks old CD1 nude mice. Non-invasive bioluminescence imaging (BLI) scan was performed using IVIS Spectrum (Perkin Elmer) after injecting 100 µl D-luciferin substrate (30 mg/ml). Mice were maintained under 2% isoflurane gas anaesthesia during the scan. The Average Radiance, represented as photons/sec/cm2/super-pixel, corresponding to the tumourigenic burden was quantified using LivingImage software v4.4. Mice were sacrificed after 24-29 days using cervical dislocation, major organs were harvested and imaged *ex-vivo* to study metastatic burden.

Hematoxylin and Eosin staining:

The staining was performed according to standard protocol for cryo sectioned tissue.

Statistical analysis

Details regarding the sample size and statistical power for each experiment are either indicated in the figure panels or described under the corresponding sections in the Methods. All data

represent results from at least three independent biological replicates, with the exact number of replicates specified in the figure legends. For quantitative analyses, mean values and standard errors were calculated. Statistical significance was primarily assessed using two-tailed Student's t-tests in GraphPad Prism 5, unless stated otherwise. P-values were determined through pairwise comparisons with the respective wild-type controls and are noted in the figure legends.

Antibodies used

Sl. No.	Name	Catalogue no.	Manufacturer
1.	MCJ (DnaJC15)	NBP2-67439	Novus
2.	Tim23	PA5-71877	ThermoFisher Scientific
3.	PGC1α	2178S	Cell Signaling Technology
4.	pAMPKα	2535S	Cell Signaling Technology
5.	HKII	sc-374091	Santa Cruz Biotechnology
6.	p65NFκβ	710048	ThermoFisher Scientific
7.	Ki67	AB16667	Abcam
8.	SDHα	459200	ThermoFisher Scientific
9.	mTOR	ERP3909(N)	Abcam
10.	E-Cadherin	ab40772	Abcam
11.	pACC2^{Ser79}	PA5-17725	ThermoFisher Scientific
12.	IDH	ab172964	Abcam
13.	β-actin	A3854	Sigma-Aldrich (Merck)
14.	Anti-OXPHOS	45-8199	ThermoFisher Scientific
15	Cpt1	MA5-51291	ThermoFisher Scientific

Primers used:

	Gene	Forward	Reverse
1	ACLY	TCGGCCAAGGCAATTTCAGAG	CGAGCATACTTGAACCGATTCT
2	FASN	AAGGACCTGTCTAGGTTTGATGC	TGGCTTCATAGGTGACTTCCA
3	CMYC	GGCTCCGGCAAAAGGTCA	CTGCGTAGTTGTGCTGATGT
4	CDK-1	GCTGCGAAGTGGAAACCATC	CCTCCTTCTGCACACATTTGAA
5	CPT1	CTGAGTCATGCGACTTCGTG	GCGAGGCGATAGATATGCTG
6	ACC2	CATGGCAAGAGAAAAGCGGC	ACTCTTGGTGATCGGCTTGG
7	DnaJC15 gRNA	G*U*C*GCUACGCAUUUCGGAUC+ Modified scaffold	U*A*G*CGCAAACUCUCGCCAAC+ Modified scaffold
